# Pangenome-level analysis of nucleoid-associated proteins in the *Acidithiobacillia* class: insights into their functional roles in mobile genetic elements biology

**DOI:** 10.3389/fmicb.2023.1271138

**Published:** 2023-09-25

**Authors:** Simón Beard, Ana Moya-Beltrán, Danitza Silva-García, Cesar Valenzuela, Tomás Pérez-Acle, Alejandra Loyola, Raquel Quatrini

**Affiliations:** ^1^Centro Científico y Tecnológico de Excelencia Ciencia & Vida, Fundación Ciencia & Vida, Santiago, Chile; ^2^Facultad de Medicina y Ciencia, Universidad San Sebastián, Santiago, Chile; ^3^Facultad de Ingeniería, Arquitectura y Diseño, Universidad San Sebastián, Santiago, Chile

**Keywords:** *Acidithiobacillus*, NAPs, plasmids, core proteins, FIS, SMC, IHF, HU

## Abstract

Mobile genetic elements (MGEs) are relevant agents in bacterial adaptation and evolutionary diversification. Stable appropriation of these DNA elements depends on host factors, among which are the nucleoid-associated proteins (NAPs). NAPs are highly abundant proteins that bind and bend DNA, altering its topology and folding, thus affecting all known cellular DNA processes from replication to expression. Even though NAP coding genes are found in most prokaryotic genomes, their functions in host chromosome biology and xenogeneic silencing are only known for a few NAP families. Less is known about the occurrence, abundance, and roles of MGE-encoded NAPs in foreign elements establishment and mobility. In this study, we used a combination of comparative genomics and phylogenetic strategies to gain insights into the diversity, distribution, and functional roles of NAPs within the class *Acidithiobacillia* with a special focus on their role in MGE biology. *Acidithiobacillia* class members are aerobic, chemolithoautotrophic, acidophilic sulfur-oxidizers, encompassing substantial genotypic diversity attributable to MGEs. Our search for NAP protein families (PFs) in more than 90 genomes of the different species that conform the class, revealed the presence of 1,197 proteins pertaining to 12 different NAP families, with differential occurrence and conservation across species. Pangenome-level analysis revealed 6 core NAP PFs that were highly conserved across the class, some of which also existed as variant forms of scattered occurrence, in addition to NAPs of taxa-restricted distribution. Core NAPs identified are reckoned as essential based on the conservation of genomic context and phylogenetic signals. In turn, various highly diversified NAPs pertaining to the flexible gene complement of the class, were found to be encoded in known plasmids or, larger integrated MGEs or, present in genomic loci associated with MGE-hallmark genes, pointing to their role in the stabilization/maintenance of these elements in strains and species with larger genomes. Both core and flexible NAPs identified proved valuable as markers, the former accurately recapitulating the phylogeny of the class, and the later, as seed in the bioinformatic identification of novel episomal and integrated mobile elements.

## 1. Introduction

The lack of a membrane-delimited nucleus is the unifying characteristic of prokaryotes, yet inside the bacterial cell, DNA is stored in a highly condensed and functionally organized nucleoid. The bacterial nucleoid 3D-structure is determined by several interconnected factors, including DNA supercoiling, curvature and bends induced by Brownian motion, the binding of nucleoid associated RNAs, proteins, and macrodomain determining proteins (e.g., SMC), in addition to gene expression patterns (Lioy et al., 2018; Verma et al., 2019). Nucleoid-associated proteins (NAPs) are numerous and diverse, and the genes encoding them are found in almost all prokaryotic genomes (Dillon and Dorman, 2010; Driessen and Dame, 2011). A distinctive characteristic of NAPs is their ability to bind DNA in both specific and non-sequence specific manners. Specific binding of NAPs to target DNA sequences is mostly involved in transcriptional regulation, DNA replication, recombination, and repair. In turn, the sequence independent binding mode of NAPs is crucial for chromosome compaction by bridging, wrapping, and bunching nearby or distant chromosome DNA segments, and also for constraining negative supercoiling (Dorman, 2013; Verma et al., 2019).

Model bacteria, *Escherichia coli*, encodes in its genome a total of 12 different NAPs (Azam and Ishihama, 1999), being the histone-like nucleoid-structuring protein H-NS (Grainger, 2016), the factor-for-inversion stimulation Fis (Finkel and Johnson, 1992), the Histone-like protein HU (Kamashev et al., 2017), and the integration host factor IHF (Lee et al., 2015), the most thoroughly studied families. While H-NS binds DNA non-specifically, preferring AT-rich and curved regions, to control gene regulation, xenogeneic silencing, and nucleoid architecture (Rimsky, 2004; Dorman, 2007), Fis binds a specific AT-rich DNA consensus sequence, to bend the DNA at the binding site and exert its effect on global transcription and chromosome organization (Duprey et al., 2014). In contrast, ubiquitous HU and closely related IHF proteins, recognize binding sites through structural and topological cues, and play a role in DNA packaging, repair, and recombination-dependent processes (Hammel et al., 2016). Given the influence of NAPs in DNA transactions that are both essential to many cellular functions and relevant for the stable appropriation of foreign DNA, understanding their diversity, species-specific distribution and genomic location seem to be key to dissect their specific contributions to cellular function in prokaryotes. Recent studies on the distribution of NAP families in bacterial genomes have shown that most species do not encode in their genomes homologs of all 12 NAPs found in *E. coli*, being only HU, Fis, IHF and EbfC ubiquitous in bacteria (Perez-Rueda and Ibarra, 2015; Perez-Rueda et al., 2018). Rather, the distribution of some NAP families in prokaryotic genomes seems to be characteristic of specific bacterial groups, as shown for H-NS-like proteins, MvaT-like, Lsr2-like and Rok-like proteins (Perez-Rueda and Ibarra, 2015). In this respect, few efforts have been placed in understanding the taxa-specific gene complement of NAPs or their phyletic patterns.

Horizontal gene transfer (HGT) is one of the major mechanisms underlying the evolution of gene content in prokaryotes (Arnold et al., 2022). Gene flow between and within microbes is driven by the “mobilome,” a vast and highly diversified repertoire of Mobile Genetic Elements (MGEs) (Weltzer and Wall, 2023). These DNA-based exchange platforms capable of self-movement can be classified according to the molecular mechanism underlying their physical movement as “translocative elements” (transposons and insertion sequences) or “integrative elements” (integrons and temperate viruses), whereas plasmids and genomic islands, are reckoned as dispersive or transmissible elements and are further classified as “conjugative” or “mobilizable” (Beard et al., 2021). Given that HGT and integration of novel genes is a routine event in most bacterial populations, the adaptability of the nucleoid architecture is a universal prerequisite. It has been proposed that NAP-induced regional differences in the chromosome’s topology may lead to unequal access of chromosomal regions to mutagenic and recombination processes, and/or modulate transcription of foreign DNA, therefore exerting a great influence on the nucleoid’s potential to evolve (Dorman, 2013; Kivisaar, 2020). Several NAPs coded in episomal and integrated MGEs have been described, which are able to influence both positively and negatively MGE recombination dynamics, maintenance in the integrated state, and MGE dispersal to suitable hosts (Flores-Ríos et al., 2019b), highlighting the potential role of NAPs in MGEs biology. However, it is only in the case of the enterobacterial H-NS-like proteins, that an irrefutable role as xenogeneic-DNA silencers has been demonstrated (Ding et al., 2015; Singh et al., 2016). Although, the role of plasmid-encoded NAPs in the regulation of the host’s and the plasmid’s transcriptional networks has been studied over the past decades in a few different bacteria (e.g., Baños et al., 2009; Yun et al., 2016), scarce evidence supports the distinction between endogenous NAPs and those linked to the mobilome as a path to dissect their specific roles (e.g., Müller et al., 2010; Shintani et al., 2015). We decided to take this path to study the role of NAPs in our model bacteria, the acidithiobacilli.

The class *Acidithiobacillia* comprises Gram-negative chemolithoautotrophic and obligate acidophilic bacteria that obtain their energy from reduced sulfur compounds oxidation using oxygen as electron acceptor. The class pertains to the phylum Proteobacteria (Williams and Kelly, 2013) and contains a single order and two families, the *Acidithiobacillaceae* with 10 validly described species (Boden and Hutt, 2019a), and the more ancestral *Thermithiobacillaceae* with only two acknowledged species (Boden and Hutt, 2019b). Extensive genomic comparisons of sequenced members of the class have revealed important lacunae in our comprehension of the phylogenetic space occupied by the class (Nuñez et al., 2017) and expanded its genomic contours to an increasing total of 19 lineages (Moya-Beltrán et al., 2021, 2023). These linages rank at different taxonomic levels, including three novel genera named as ‘*Fervidacidithiobacillus*’ (ex. *Acidithiobacillus caldus*), ‘*Igneacidithiobacillus*’, and ‘*Ambacidithiobacillus*’ (*Acidithiobacillus sulfuriphilus*), in addition to the two long acknowledged genera *Thermithiobacillus* and *Acidithiobacillus* (Kelly and Wood, 2000). With the exception of the *Acidithiobacillus*, which includes both sulfur-and iron-oxidizing species (i.e., it is a “dual physiology” clade), the other 4 genera are exclusively sulfur-oxidizers (Moya-Beltrán et al., 2021). Despite several common characteristics, the class members are highly heterogeneous, and evolutionary trajectories defining their ancestry and differentiating characteristics are only beginning to be understood.

Members of the class are known to carry a variety of MGEs, these being an important source of genomic and phenotypic diversity. Some MGEs described in *Acidithiobacillia* are: (i) small cryptic plasmids found in strains of *Acidithiobacillus ferrooxidans*, *Acidithiobacillus ferridurans* and ‘*Fervidacidithiobacillus caldus*’ (e.g., Chakravarty et al., 1995; Dominy et al., 1998), (ii) mobilizable plasmids found in ‘*F. caldus*’ and iron oxidizing *Acidithiobacillus* spp. (e.g., Rawlings, 2005; Tran et al., 2017), (iii) several genomic islands in *A. ferrooxidans* and ‘*F. caldus*’ (e.g., Orellana and Jerez, 2011; Acuña et al., 2013), (iv) and a plethora of integrative or translocative mobile elements such as insertion sequences (IS) and transposons yet to be characterized (e.g., Holmes et al., 2001). A number of NAP-like genes have been reported during the annotation of plasmids, genomic islands and other MGEs in *Acidithiobacillus* species genomes (Rawlings, 2005; Bustamante et al., 2012; Flores-Ríos et al., 2019a). Despite their frequent identification, little is known about the diversity, distribution, and functional roles of endogenous and MGE-associated NAPs within the class *Acidithiobacillia*.

In this study, we used the emerging phylogenetic structure of the *Acidithiobacillia* class as a guide to conduct comparative genomics and phylogenetic analysis of NAPs. Our goal was to gain insights into the role of NAPs in maintaining nucleoid architecture and function, across species of the class with different lifestyles and occupying distinct econiches, and to assess their relevance in MGE biology, and therefore in genomic evolution and diversification within the *Acidithiobacillia* class. To this end we performed a systematic search of known NAP protein families in the genomes of 93 different *Acidithiobacillia* species strains, and then mapped the resulting NAP pool to the core versus flexible gene complements of the different genera and species. We also assessed their occurrence in chromosomal loci versus episomal and integrated MGEs. By performing phylogenetic analysis of the identified NAPs, and analyzing their genomic contexts, we gained insights into their essentiality, diversification and potential roles, while also revealing their value as phylogenetic and MGE-specific markers.

## 2. Materials and methods

### 2.1. Genomes and query profiles

A total of 93 complete and draft genomes corresponding to different *Acidithiobacillia* class species were obtained from the public WGS NCBI Genome database. Drafts were assessed for contamination and completeness as previously reported (Moya-Beltrán et al., 2019, 2021). The complete list of genomes used in this study, their assembly statistics, along with relevant metadata can be found in Supplementary Table S1. Models selected to profile NAP protein families in the *Acidithiobacillia* included Alba, CbpA, Csp., Dps, EbfC, Fis, Hha, YmoA, H-NS, StpA, MvaT, HU, IHF, HupB, Lrp, Lsr2, MukB, YccC and YejK. In addition to the aforementioned NAPs, a model for the plasmid replication protein KfrA was also included as plasmid-specific NAP (Jagura-Burdzy and Thomas, 1992). The full list of models/profiles used in this study can be found in Supplementary Table S2. Multiple sequence alignments of the protein families of interest, to be used as queries in the identification of NAPs-related conserved domains in candidate protein sequences, were downloaded from the conserved domain database (Marchler-Bauer et al., 2017; CDD v3.17, downloaded on 04/01/2019). The CD database included the domain models and/or profiles from Pfam, COG, TIGRFAMs, and SMART databases (Tatusov et al., 2000; Haft et al., 2013; Finn et al., 2016; Letunic and Bork, 2018).

### 2.2. Candidate NAPs gene identification, functional assignment and curation

NAP proteins present in the *Acidithiobacillia* class CDS database were identified using the workflow presented in Supplementary Figure S1. Briefly, multiple sequence alignments of the protein families of interest listed in Supplementary Table S2, were used as queries to search each genome of the class using PSI-BLAST (Altschul et al., 1997) (five iterations; statistical significance established at *e*-value 0.01). Candidate target sequence assignments were validated against the CDD using CDsearch (Marchler-Bauer et al., 2017) and hhsearch (Fidler et al., 2016), with defaults parameters (*e*-value <10E-5 for CD-search and hhsearch and probability >90% for hhsearch). Identification of specific domains in selected proteins was done with RPS-BLAST v2.2.26 versus CDD v3.17. Elimination of overlapping motifs and redundant results were done with rpsbproc v0.11. Orthology was determined as reported previously (Moya-Beltrán et al., 2021). Briefly, COGtriangles (v2.1) was used as clustering algorithm and triangle reciprocal hits were clustered in Protein Families (PFs). BLAST pairwise alignment cutoffs were set at 75% coverage and e-values at 10E-5. Selected proteins were clustered by sequence identity (threshold of 50% and defaults parameters) with Usearch v1.2.22 (Edgar, 2010). Protein alignments for the candidate NAPs recovered for the *Acidithiobacilla* were constructed with the MAFFT v7.453 program (Katoh and Standley, 2013). Selected clusters and proteins were re-annotated and curated manually, when required.

### 2.3. General data analysis and visualization of results

Molecular weight and theoretical pI (isoelectric point) of candidate proteins were computed through the Pepstats software included in the EMBL-EBI search and sequence analysis tools.^1^ To analyze NAPs vicinities, CDS encoded up to 5 kbp up- and downstream from each candidate NAP were identified and their functional annotations were retrieved using DIAMOND blast search (Buchfink et al., 2015) against the KEGG database (Kanehisa et al., 2016). A frequency index (KEGG-based vicinity frequency index) was then derived for each annotated CDS in the NAPs vicinities. For any given NAP family, the percentage of NAP genes with a given CDS in its vicinity was scored and the frequency expressed as a percentage of the total number of NAP-encoding genes in the family. Genetic context and clustering visualization were conducted using Clinker v0.0.20 program (Gilchrist and Chooi, 2021) or the R package gggenes.^2^ Inverted repeats were identified by using IUPACpal (Alamro et al., 2021) and promoter and transcriptional regulators binding sites were predicted by using BPROM at Softberry^3^ and Virtual Footprint at Prodoric (Dudek and Jahn, 2022)^4^ websites. Data analysis and visualization were conducted using the R language (R version 3.6.3) with tidyverse v1.3.0 (Wickham et al., 2019) and ggplot2 v3.3.2 (Wickham, 2016) packages. Circular genome representation was done with Circos (Krzywinski et al., 2009).

### 2.4. Comparative genomics and phylogenetic analysis

Genome comparisons were performed using the GET_HOMOLOGUES software package v3.3.2 (Contreras-Moreira and Vinuesa, 2013) and orthology was determined using COGtriangles (v2.1) as clustering algorithm and triangle reciprocal hits were clustered in Protein Families (PFs). Given that draft genomes and MAGs are included in the comparison, in this study we used a permissive threshold of 90% to define the core protein family sets (i.e., PFs with representatives in more than 90% of the genomes analyzed). To aid in NAP content comparisons, we defined a *frequency index* and a *paralogue index*. The *frequency index* was calculated for each NAP protein family as the ratio of genomes for every given *Acidithiobacillia* lineage that encodes candidate NAPs of that protein family divided by the total number of genomes of that given lineage. A threshold of 0.90 was set empirically, to discriminate between *conserved* (frequency > 0.90) and *non-conserved* (frequency ≤ 0.90) PFs, based on both lineage-level occurrence and PF clusters coherence. The *paralogue index* was calculated for each NAP protein family as the ratio of the average genetic dose of NAP coding genes to the number of different NAP PFs for every given *Acidithiobacillia* lineage that encodes candidate NAPs of that protein family. Multiple sequence alignments were constructed with MAFFT v7.453 program (Katoh and Standley, 2013) and edited using Jalwiew (Waterhouse et al., 2009). The FastTree program v2.1.10 (WAG evolutionary model for amino acid sequences analyses) with bootstrap resampling (1,000 replicates) (Price et al., 2010) and MEGA X (Kumar et al., 2018) were used for phylogenetic analysis. FigTree v1.4.4^5^ was used for tree visualization and manipulation.

### 2.5. Identification of mobile genetic elements and MGE hallmark genes

Putative mobile genetic elements present in the queried genomes were identified using an integrative approach, as described in González et al. (2014). Identification of MGEs hallmark genes in complete and draft genomes was carried out using the following programs: CONJscan for Type IV secretion systems prediction (Guglielmini et al., 2014) and TnpPred for prediction of prokaryotic transposases (Riadi et al., 2012). When needed, contigs were aligned and reordered to a reference genome by using the Mauve software (Darling et al., 2004). To identify plasmid encoded NAPs, we used a customized protein repository consisting of 1,388 CDS retrieved from public *Acidithiobacillia* plasmid sequences and refined their annotations and functional assignments as described above.

### 2.6. RNA-seq data analysis

RNA-seq expression profiles for *Acidithiobacillus thiooxidans* type strain ATCC 19377 grown in elemental sulfur at pH 2.5 at 30°C (Camacho et al., 2020) (BioProject PRJNA541131) were downloaded from SRA database.^6^ Selected runs (SRR9016879, SRR9016878, and SRR9016873) were aligned to *A. thiooxidans* ATCC 19377 reference genome (NZ_CP045571) using bowtie2 v2.5.0 (Langmead and Salzberg, 2012) and samtools v1.10 (Danecek et al., 2021) for bam files processing and visualization. To assess relative RNA-seq expression levels, depth coverage were calculated for the whole *A. thiooxidans* genome sequence as the number of reads per bin (10 base-long) and normalized by reads per genomic content (RPGC, 1x normalization) using the bamCoverage tool from deepTools v2.0 library (Ramírez et al., 2016). RPGC (per bin) = number of reads per bin/scaling factor for 1x average coverage. RNA-seq coverage RPGC were obtained using extended paired RNA-seq reads and an effective genome size of 3,415,726 bases.

## 3. Results and discussion

### 3.1. General overview of identified *Acidithiobacillia* class candidate NAPs

To gain insights into the distribution and functional roles of NAPs within the *Acidithiobacillia* class, we analyzed 93 genomes belonging to 10 validated and 7 candidate species (named as proposed in Moya-Beltrán et al., 2021), comprising taxa of varied physiological characteristics and lifestyles (Supplementary Table S1). The genomes were searched for CDSs containing NAP domains (Supplementary Table S2), according to the *in silico* analysis workflow schematized in Supplementary Figure S1. A total of 1,197 candidate CDSs were recovered and manually classified into 12 different NAP families (Alba_2, EbfC, Fis, H-NS, HU, IHF_A, IHF_B, KfrA, Lrp, MukB, NdpA and SMC) according to their CDD/hmms-based inferred functional assignment (Table 1). Detailed results obtained are presented in Supplementary Table S3.

**Table 1:** Occurrence and abundance of NAP proteins in *Acidithiobacillia* class bacteria.

Species name^#^	Total NAPs	NAPs/Genome		Total proteins (range per genome)																							
		Median	Range	EbfC		Fis		H-NS		HU		IHF_A		IHF_B		Lrp		SMC		Alba_2		KfrA		NdpA		MukB	
*Acidithiobacillus ferrooxidans* [AFE] (*n* = 14)	195	15	10–17	14 (1)		13 (1)		0 (0)		43 (2–5)		58 (2–6)		22 (1–3)		14 (1)		15 (1–2)		11 (1)		5 (1–2)		0 (0)		0 (0)	
*Acidithiobacillus ferruginosus* [AFG] (*n* = 1)	12	12	12	1 (1)		1 (1)		0 (0)		3 (3)		2 (2)		1 (1)		1 (1)		1 (1)		0 (0)		2 (2)		0 (0)		0 (0)	
*Acidithiobacillus ferridurans* [AFD] (*n* = 9)	134	15	11–17	9 (1)		9 (1)		1 (1)		43 (3–6)		31 (2–5)		14 (1–2)		9 (1)		9 (1)		2 (1)		7 (1–2)		0 (0)		0 (0)	
*Acidithiobacillus ferriphilus* [AFP] (*n* = 9)	86	10	6–12	9 (1)		9 (1)		1 (1)		18 (1–3)		18 (1–3)		11 (1–2)		8 (1)		10 (1–2)		0 (0)		2 (1)		0 (0)		0 (0)	
*Acidithiobacillus ferrivorans* [AFV] (*n* = 7)	115	16	13–25	7 (1)		7 (1)		6 (1)		18 (1–4)		35 (3–9)		20 (1–7)		9 (1–2)		7 (1)		1 (1)		5 (1–2)		0 (0)		0 (0)	
*Acidithiobacillus ferianus* [AFN] (*n* = 1)	8	8	8	1 (1)		1 (1)		0 (0)		1 (1)		1 (1)		1 (1)		2 (2)		1 (1)		0 (0)		0 (0)		0 (0)		0 (0)	
*Acidithiobacillus thiooxidans* [ATH] (*n* = 21)	352	17	11–23	20 (1)		20 (1)		36 (1–3)		91 (1–7)		77 (2–5)		62 (2–6)		21 (1)		21 (1)		0 (0)		1 (1)		0 (0)	3 (1)	
*`Acidithiobacillus concretivorus * [ACO] (*n* = 1)	14	14	14	1 (1)		1 (1)		1 (1)		4 (4)		3 (3)		2 (2)		1 (1)		1 (1)		0 (0)		0 (0)		0 (0)		0 (0)	
*`Acidithiobacillus sulfurivorans * [ASU] (*n* = 1)	17	17	17	1 (1)		1 (1)		0 (0)		5 (5)		2 (2)		5 (5)		1 (1)		1 (1)		0 (0)		0 (0)		1 (1)		0 (0)	
*`Acidithiobacillus marinus * [AMA] (*n* = 1)	9	9	9	1 (1)		1 (1)		0 (0)		2 (2)		1 (1)		1 (1)		1 (1)		1 (1)		1 (1)		0 (0)		0 (0)		0 (0)	
*`Acidithiobacillus monserratensis * [AMO] (*n* = 1)	10	10	10	1 (1)		1 (1)		0 (0)		1 (1)		1 (1)		3 (3)		1 (1)		1 (1)		0 (0)		1 (1)		0 (0)		0 (0)	
*`Fervidacidithiobacillus caldus * [FCA] (*n* = 18)	189	10	7–14	18 (1)		18 (1)		0 (0)		42 (1–5)		55 (1–5)		21 (1–2)		0 (0)		18 (1–2)		0 (0)		17 (1–2)		0 (0)		0 (0)	
*`Igneacidithiobacillus copahuensis * [ICO] (*n* = 6)	38	6	6–7	6 (1)		6 (1)		0 (0)		6 (1)		6 (1)		6 (1)		0 (0)		6 (1)		0 (0)		2 (1)		0 (0)		0 (0)	
*`Igneacidithiobacillus yellowstonensis * [IYE] (*n* = 1)	5	5	5	1 (1)		1 (1)		0 (0)		1 (1)		1 (1)		0 (0)		0 (0)		1 (1)		0 (0)		0 0)		0 (0)		0 (0)	
*`Ambacidithiobacillus sulfuriphilus * [AMS] (*n* = 1)	7	7	7	1 (1)		1 (1)		0 (0)		1 (1)		1 (1)		1 (1)		1 (1)		1 (1)		0 (0)		0 (0)		0 (0)		0 (0)	
*Thermithiobacillus tepidarius* [TTP] (*n* = 1)	6	6	6	1 (1)		1 (1)		0 (0)		1 (1)		1 (1)		1 (1)		0 (0)		1 (1)		0 (0)		0 (0)		0 (0)		0 (0)
(*n* = 93)	1,197			92	91	45	280	293	171	69	95	15	42	1		3	

^#^The acronym for each species is provided within brakets as proposed in Moya-Beltrán et al. (2021). The number of genomes analyzed per lineage (n) is shown in parenthesis.

**Figure 1:**
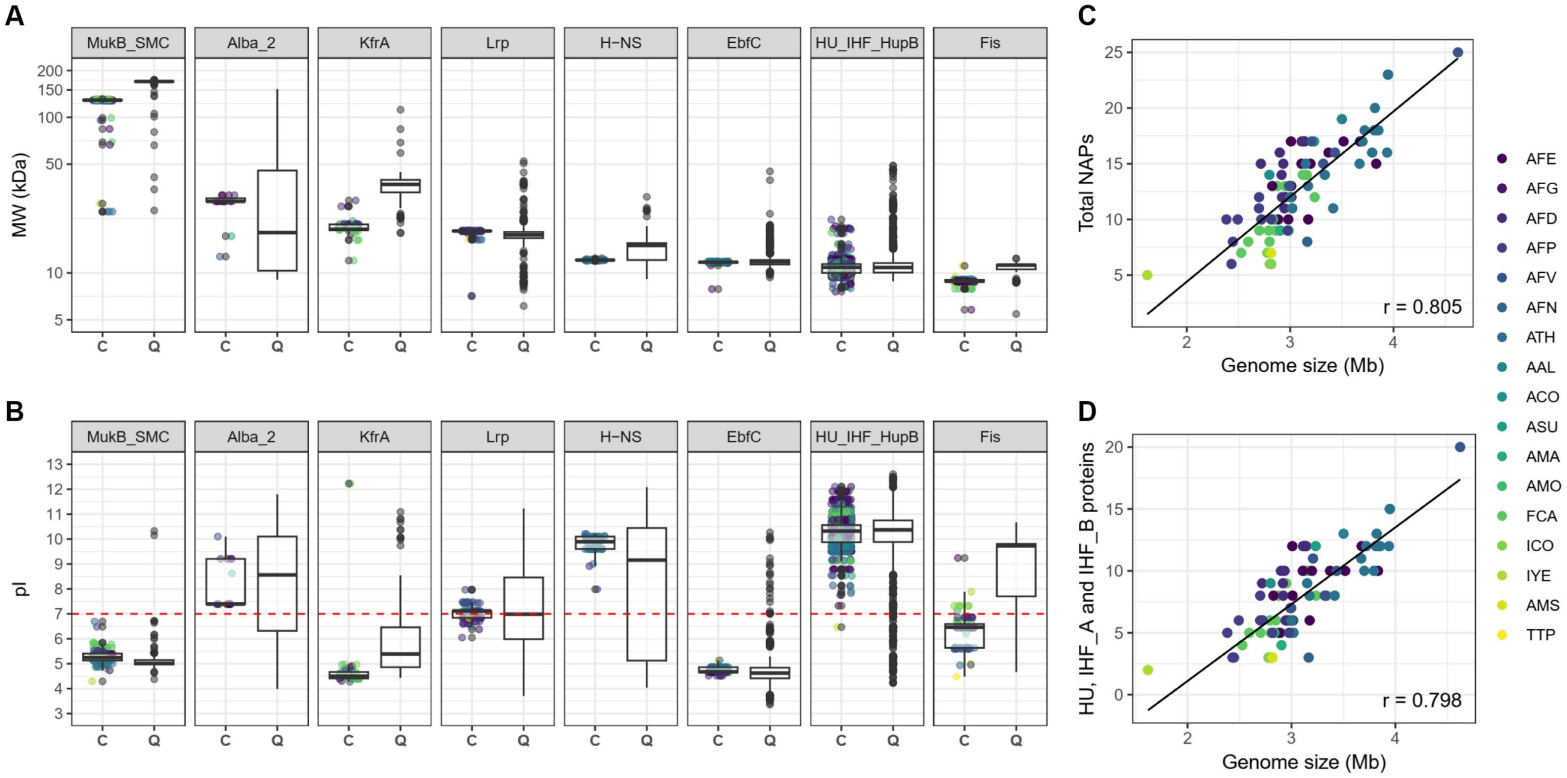
Predicted physicochemical properties and correlations of candidate NAPs abundance versus genome size in *Acidithiobacillia* class representatives. Predicted molecular weight in KDa **(A)** and isoelectric point **(B)** for the different candidate *Aciditiobacillia* NAP families identified in this study compared to the proteins of the CD database used as queries. Correlation between the total number of NAP types **(C)** or between the number of HU and IHF NAPs **(D)** and genome size in Kb. Pearson’s correlation coefficient (*r*) is shown at bottom right of each scatter plot. In **(A,B)** *C* stands for candidate and *Q* for query. AFE, *Acidithiobacillus* *ferrooxidans* AFG, ‘*Acidithiobacillus*’ *ferruginosus*; AFD, *Acidithiobacillus ferridurans*; AFP, *Acidithiobacillus ferriphilus*; AFV, *Acidithiobacillus ferrivorans*; AFN, *Acidithiobacillus ferrianus*; ATH, *Acidithiobacillus thiooxidans*; AAL, *A. thiooxidans* subsp. *albertensis*; ACO, *Acidithiobacillus concretivorus*, ASU, ‘*Acidithiobacillus sulfurivorans*’; AMA, ‘*Acidithiobacillus marinus*’; AMO, ‘*Acidithiobacillus*’ *monserratensis*; FCA, ‘*Fervidacidithiobacillus caldus*’; ICO, ‘*Igneacidithiobacillus copahuensis*’; IYE, ‘*Igneacidithiobacillus yellowstonensis*’; AMS, ‘*Ambacidithiobacillus sulfuriphilus*’; and TTP, *Thermithiobacillus tepidarius*.

Predicted molecular weights (MW) and isoelectric points (pI) of the candidate *Acidithiobacillia* NAPs were compared to those of the CDD proteins used as queries (Figures 1A,B). General agreement in both MW and pI was found between both datasets. However, orthologs encoding candidate Alba_2 showed a higher median MW, whereas candidate MukB/SMC, KfrA, H-NS and Fis proteins were smaller than the median queries outside *Acidithiobacillia*. In accordance with canonical bacterial NAPs (Dillon and Dorman, 2010), candidate Lrp, H-NS, EbfC, HU/IHF/HupB and Fis proteins were small (<18,59 kDa), single-domain proteins. Among the later HU, IHF_A, IHF_B and H-NS orthologs were also predicted as basic proteins (theoretical pI >9.66), while candidate Fis had a more acidic theoretical median pI (median pI of 6.46), compared to CDD proteins used as queries (with median pI of 9.44). Given that the net charge of the potein at physiological pH should be relevant in Fis DNA-binding affinity, we inspected this trend more closely in a subset of single-domain Fis proteins from several known acidophiles outside the class (Supplementary Figure S2).

Invariantly, all extreme acidophilic genera analysed (with optimal growth pH < 3.0), had Fis proteins with median pI below 8, while moderate acidophiles (with optimal growth at pH < 4.5) exceeded this value. Intriguingly, a positive correlation between the increase in the median pI of the predicted Fis proteins and an increase in the optimum pH of growth (Pearson correlation coefficient *r = 0.683*), was observed in both among extreme (e.g., *Acidiferrobacter, Acidihalobacter, Ferrovum* and *Acidithiobacillia* spp.) and moderate acidophiles (e.g., *Thiomonas, Acidobacteria* and *Silvibaterium* spp.). In contrast, in genera with only mild acid tolerance (having reported optimal growth at pH < 6.0), including *Acetobacter,* some *Acidiphilium* strains*, Gallionella* and *Methylocella*, a negative correlation between median Fis pI and optimal growth pH was obtained (Pearson correlation coefficient *r* = −0.586). The observed values and the co-variation of the pI and the optimal pH of growth in true acidophiles, suggests that the more acidic pI of Fis orthologs is favoured in this group of organisms to ensure a slightly positive net charge for this DNA-binding protein at cytoplasmatic pH (pH 6.0–7.0; Ingledew, 1982). Such positive charge could facilitate electrostatic interactions with the negatively charged DNA backbone, potentially enhancing DNA binding and specificity. This feature of Fis proteins could reflect a particular adaptation of these group of microorganisms to thrive in their low pH environment and/or to endure transient pH fluctuations. Adaptative adjustments of protein’s median pI values have been linked to the acidophilic lifestyle before. Acid-exposed periplasmic and outer membrane proteins have been proven to have more basic pI values compared to neutrophiles and alkaliphiles counterparts, reflecting structural adaptations linked to the stabilization of proteins at very low pH (Chi et al., 2007).

The most abundant NAP families encoded in *Acidithiobacillia* genomes are IHF_A, HU and IHF_B families, representing, respectively, 24.5, 23.4, and 14.3% of the total NAPs identified. The median number of NAPs encoded per genome in the *Acidithiobacillia* class was 10 (Table 1), and ranged between 5 in ‘*Igneacidithiobacillus yellowstonensis*’ (currently a 72.7% complete MAG, Zhou et al., 2020) and 20 in *A. thiooxidans* subsp. *albertensis* (99.9% complete, Castro et al., 2017). Deep branching *Acidithiobacillia* species *Thermithiobacillus tepidarius, `Ambacidithiobacillus sulfuriphilus * and ‘*Igneacidithiobacillus copahuensis*’ consistently had fewer NAPs per genome (97.14% complete drafts), ranging between 5 and 7. On the other hand, larger numbers of NAPs per genome were observed in the ‘*Fervidacidithiobacillus*’ and *Acidithiobacillus* species (excepting the most ancestral iron oxidizer *Acidithiobacillus ferrianus*, Table 1), supporting the expansion and/or acquisition of NAP coding genes during the diversification of the taxon. The data obtained showed a positive Pearson’s correlation coefficient between the number of NAPs and genome size (*r* = 0.805, Figure 1C), which is largely explained by the increase in the number of HU, IHF_A and IHF_B family representatives in species with larger genomes (*r* = 0.798, Figure 1D).

### 3.2. Pangenomic analysis of *Acidithiobacillia* candidate NAPs reveals the presence of class- and genus-specific sets of core proteins

**Figure 2:**
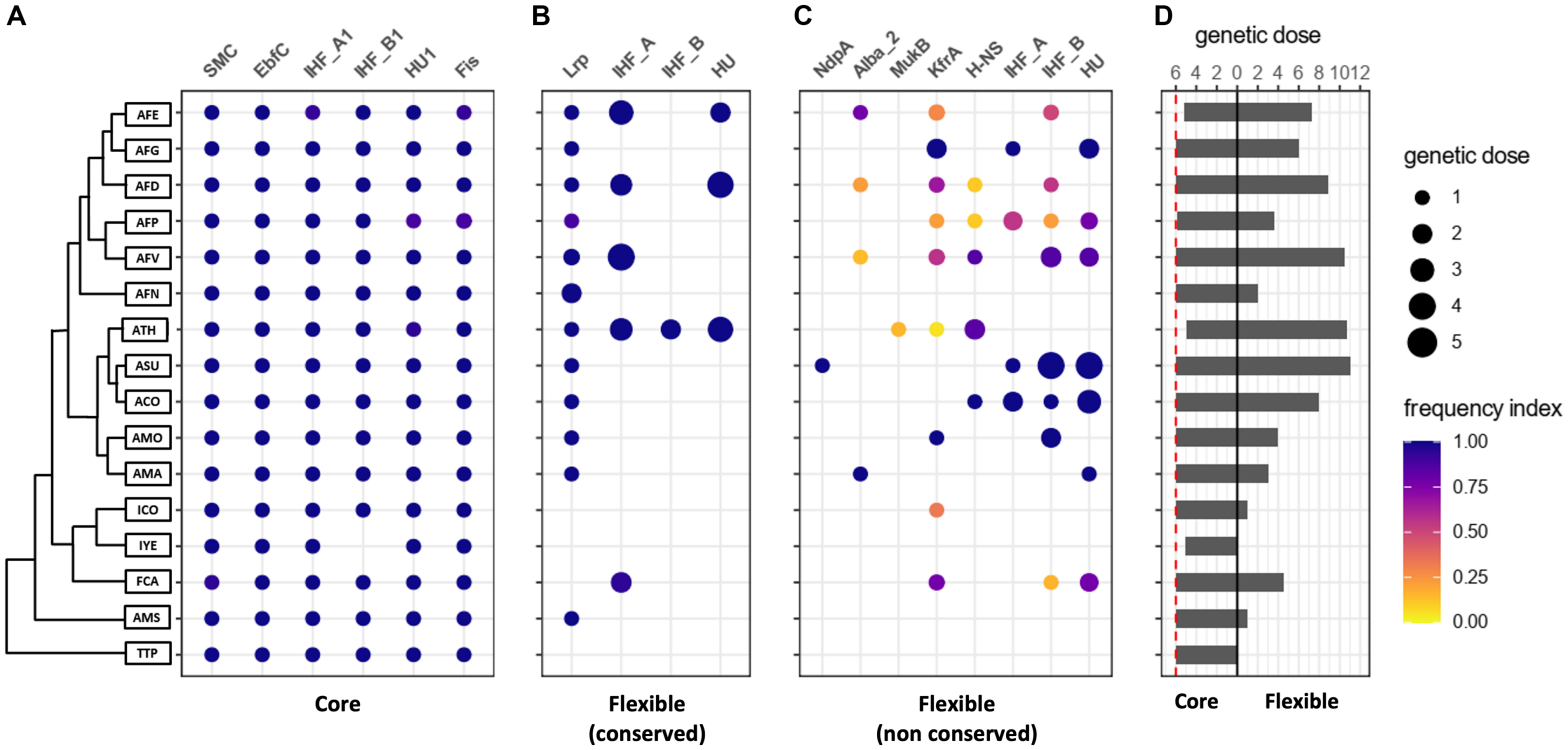
Pangenome-scale analysis of candidate NAP PFs of the *Acidithiobacillia* class. **(A)** Core NAP PFs in *Acidithiobacillia* class genomesj. **(B,C)** Flexible NAP PFs. **(D)** Average genetic dose of candidate NAP PFs in *Acidithiobacillia*, classified according to species and pangenome compartment, also represented in **(A–C)** by the size of the dots (side menu). For each lineage and NAP family pair, a *frequency* index was defined as the number of genomes in which the candidate NAP PF was found, divided by the total number of genomes of that given lineage. Dots in each panel are colored by their frequency index, according to the side bar. This index was set to discriminate between *conserved* (frequency > 0.90; **B**) and *non-conserved* (frequency ≤ 0.90; **C**) flexible pangenome sub compartments. The phylogenetic tree to the left of **(A)** is a simplified version of the Maximun Likelihood tree obtained from the concatenated alignment of 107 conserved single-copy core proteins of the *Acidithiobacillia* class, as in Moya-Beltrán et al. (2021).

To further explore *Acidithiobacillia* NAPs diversity and distribution among different representatives of the class, we undertook a pangenome-scale analysis. Candidate NAPs were clustered in Protein Families (PFs) and their membership to the core or flexible gene complements was defined. To further analyze both protein sets, we classified candidate NAP PFs according to a *frequency index* (see methods for further detail), where core NAPs were defined to have a frequency index of one (Figure 2A). Flexible pangenome NAP PFs encoded in all available genomes of a given lineage (*frequency index* ≥ 0.90) were classified as “conserved” (species-specific), and those with *frequency index* < 0.90, or encoded in *Acidithiobacillia* lineages represented by single genomes, were classified as “non-conserved” (Figures 2B,C).

Comparative genomic analysis identified a set of 6 core NAP PFs encoded by single copy genes, and highly conserved between lineage-specific strains across the class. This core set included the SMC, EbfC, Fis, HU1, IHF_A1 and IHF_B1 families (Figures 2A). Even if the distinction between chromosomally-encoded NAPs (also referred to as endogenous NAPs) and MGE-encoded NAPs is well acknowledged (reviewed in Flores-Ríos et al., 2019a), no studies have actually differentiated the NAP PFs core-sets from the flexible ones. Indirectly, the occurrence of NAPs on plasmids (e.g., Shintani et al., 2015), pathogenicity islands (e.g., Leh et al., 2017) and other types of MGEs, such as integrative conjugative elements (Flores-Ríos et al., 2019b), links some of these NAP PFs to the flexible gene complement of Proteobacteria, yet the lack of comprehensive and systematic studies addressing this issue in other bacteria prevents us from further comparing these results with those in other microorganisms.

HU, IHF_A and IHF_B family representatives were also assigned to the flexible pangenome (Figures 2C,D). Among the latter, candidate HU family NAP clusters showed gene doses ranging from one copy per genome in the deep-branching *T. tepidarius, ‘A. sulfuriphilus’’* and *‘Igneacidithiobacillus’* spp., up to 6 copies per genome in derived species such as *A. thiooxidans* strains, e.g., ATCC 21835 (Table 1). Clustering of the HU proteins based on pairwise blast identity values, revealed the presence of 3 variants of this protein family, referred to as cluster 1–3 in text and figures (Supplementary Figure S3A). HU1 variant (cluster 1) consisted of 86 proteins distributed among all *Acidithiobacillia* genomes with a genetic dose of one coding gene per genome, albeit if absent in some of the lower quality draft genomes. This variant was thus considered as core NAP in the class. This assignment was further supported by the Maximum Likelihood (ML) trees constructed using the HU1 subset (Supplementary Figure S3B), which recapitulate the proposed phylogeny of the class (Moya-Beltrán et al., 2021). Conversely, the topology of the ML tree built with the HU2 (cluster 2) subset, comprising 104 proteins, was consistent with a history of horizontal transfer (Supplementary Figure S3C). It is known that HU proteins are highly conserved in bacteria. In *E. coli* two different HU subunits have been described, encoded by two distinct genes (*hupA* and *hupB*), which give rise to both homo-and heterodimers depending on the growth-phase, being the heterodimer required for long term survival (Claret and Rouviere-Yaniv, 1997). While the heterodimeric HU conformation seems to be a characteristic feature of enterobacteria, non-enteric bacteria such as *Vibrio proteolyticus*, *Haemophilus influenzae,* and *Pseudomonas aeruginosa* form homodimers (Oberto and Rouviere-Yaniv, 1996). Identification of only one core HU protein encoded in *Acidithiobacillia* genomes supports the formation of homodimeric HU species in members of this class. Yet, more research is needed to find out if the presence of additional copies of HU proteins (belonging to flexible HU PFs) found in some *Acidithiobacillia* genomes (i.e., in 73 genomes spanning 10 species), participate in the formation of HU heterodimers, and if this could be advantageous for long term cell survival, as proposed for *E. coli* (Claret and Rouviere-Yaniv, 1997).

In addition to the NAPs shared by all *Acidithiobacillia* genomes, we found 628 candidate NAPs within the flexible gene complement of the class. This flexible NAPs repertoire is composed by 445 proteins conserved in specific *Acidithiobacillia* lineages (*frequency index* ≥ 0.90), forming a species-specific set of proteins (Figures 2B), and 183 proteins poorly conserved even at the lineage specific level (*frequency index* < 0.90), and thus forming a *non-conserved* set of NAP proteins (Figure 2C). For instance, HU3 (cluster 3) proteins fall in this category. Candidate Lrp proteins fall within the first flexible category and show a distribution that is of note (Figure 2B). Clustering analysis revealed the presence of two Lrp variants, one of which is exclusive to the *Acidithiobacillus* genus representatives. Its position, invariably divergent to the *leuA-2* gene encoding the 2-isopropylmalate synthase involved in l-leucine biosynthesis (Supplementary Figure S4A), suggests that *Acidithiobacillus* Lrp proteins play a specific rather a global regulatory role, most likely related to regulation of amino acid biosynthetic pathways (Brinkman et al., 2003). A second Lrp variant is restricted to ‘*A. sulfuriphilus*’ CJ-2, *A. ferrianus* MG and two *Acidithiobacillus ferrivorans* strains (ACH and SS3), the latter being the only *Acidithiobacillia* representatives encoding both Lrp variants. The genetic context of this second Lrp variant coding gene is less conserved, being encoded downstream a predicted sulfite exporter of the TauE/SafE family (Supplementary Figure S4B). Functional association between these two genes in four strains of at least two species of the class suggests a possible regulatory role by Lrp during transcription of the *tauE* anion transporter encoding gene, and links this regulator with sulfur compounds metabolism.

### 3.3. Gene vicinity and expression analysis provide hints into the functional role of core NAPs PFs

**Figure 3:**
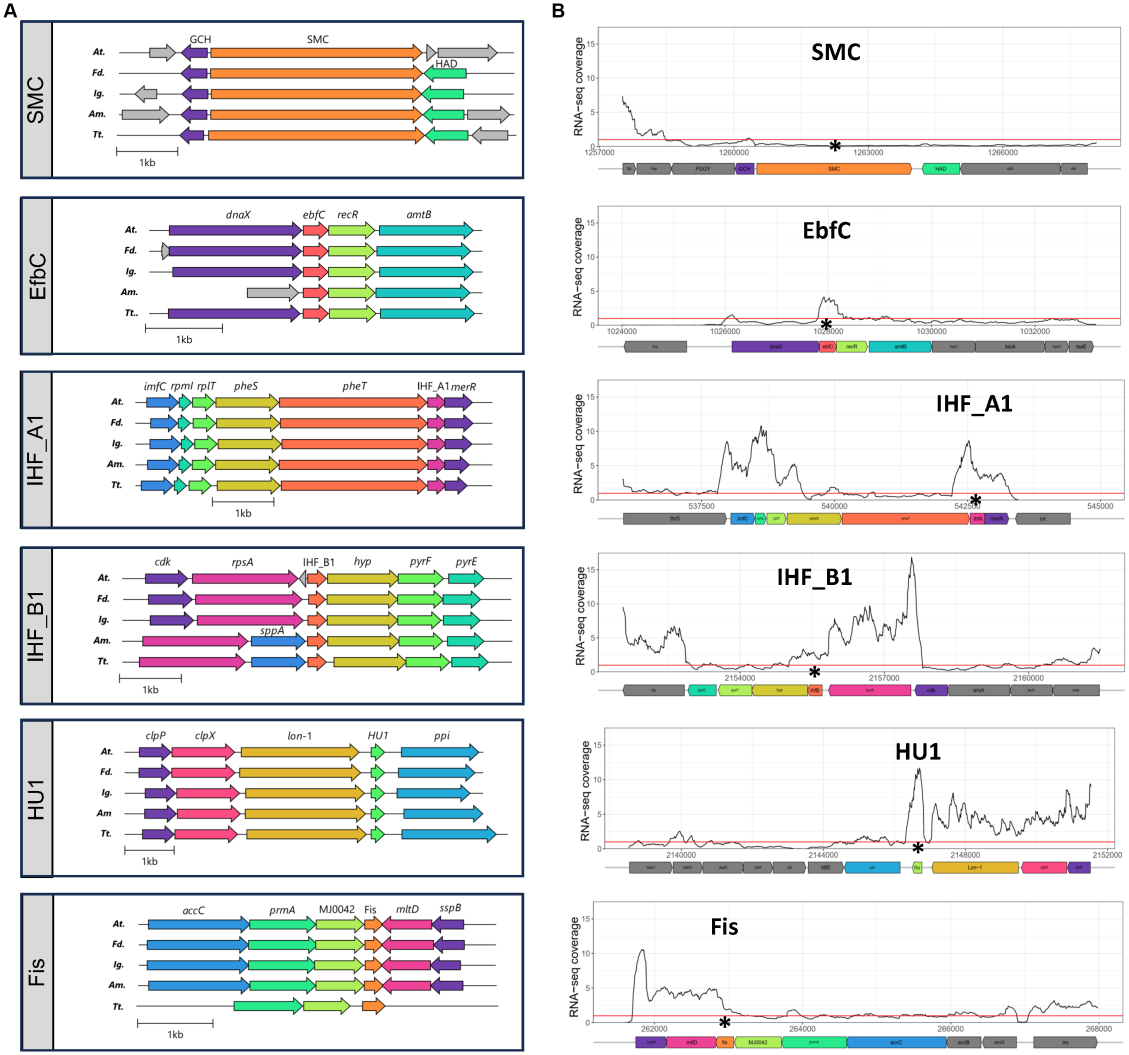
**(A)** Genetic context of core NAP PFs in representative genomes of the five genera of the *Acidithiobacillia* class: *At.*, *Acidithiobacillus* genus represented by *A. ferrooxidans* ATCC 23270^T^; *Fd.*, ‘*Fervidacidithiobacillus*’ genus represented by ‘*F. caldus*’ ATCC 51756 ^T^; *Ig.*, ‘*Igneacidithiobacillus*’ genus represented by ‘*I. copahuensis*’ VAN18-1^T^; *Am.*, ‘*Ambacidithiobacillus* ‘genus represented by ‘*A. sulfuriphilus*’ CJ-2 ^T^; and *Tt.*, *Thermithiobacillus* genus represented by *T. tepidarius* DSM 3134^T^. **(B)** Relative transcriptional expression levels of core NAP genes, and genes in their immediate vicinities, in *A. thiooxidans* type strain ATCC 19377^T^. RNA-seq expression profiles for *A. thiooxidans* ATCC 19377^T^ grown in elemental sulfur at pH 2.5 and 37°C (BioProject PRJNA541131) were downloaded from SRA database (https://www.ncbi.nlm.nih.gov/sra). Selected runs (SRR9016879, SRR9016878 and SRR9016873) were aligned to *A. thiooxidans* ATCC 19377^T^ reference genome (NZ_CP045571) and RNA-seq coverage were calculated as the number of reads per bin (10 base-long) and normalized by reads per genomic content (1x normalization). Asterisks show the location of the corresponding NAP coding gene and the red line shows the whole genome average RNA-seq coverage (defined as 1.0).

Analysis of genetic contexts around single copy core NAPs showed that their immediate vicinities were highly conserved across the *Acidithiobacillia* class members spanning the 5 known genera (Figure 3A). Core NAPs neighbouring genes were invariantly housekeeping genes, coding for essential proteins related to nucleotide (e.g., EfbC) and protein synthesis (e.g., IHF), and metabolism (e.g., Fis). Specific details of the inferred roles for SMC, MukB, EbfC, IHF, HU and Fis are provided and discussed in Supplementary Figures S4C–J (see extended figure legend). The nature and conservation of the contexts for all these core NAPs, indicated that these genes were located in genomic regions of high transcriptional activity. By extension, it is likely that these NAP-ecoding genes are also all highly expressed genes.

To test this assertion, we analysed publicly available transcriptomic data for *A. thiooxidans* type strain ATCC 19377, the type species of the *Acidithiobacillus* genus (see methods for details). All 6 core NAP proteins were expressed during exponential growth of this strain under standard culturing conditions (elemental sulfur at pH 2.5 and 30°C) (Figure 3B). Among them, and under the tested conditions, the SMC coding gene showed expression levels below the genome average. This could indicate that *smc* is transcriptionally expressed at low levels in constitutive manner, or either that it is induced only during specific stages of the cell-cycle, and thus not represented in the exponential growth culture from which the RNA-seq data originated. Evidence pointing in this direction has been obtained at least in two microorganisms, the archaeon *Halobacterium salinarum* (Herrmann and Soppa, 2002) and the bacterium *Caulobacter crescentus* (Jensen and Shapiro, 2003), where transcription of the *smc* gene has been shown to be circumscribed to early stationary phase. Further experiments using synchronous cultures would be required to test such cell-cycle dependency of *smc* transcription in *A. thiooxidans*.

All other core NAP-encoding genes were expressed at levels above the genome average (RNA-seq coverage >1.0). In particular, IHF_A1 and HU1 genes showed the highest expression levels (RNA-seq coverage up to ~9.0 and ~12.0, respectively). This is most likely due to the variety of processes in which these proteins play a part. For instance, HU proteins bind DNA in a non-sequence-specific manner, and have been proposed to prevent DNA damage under stress conditions and mediate gene expression regulation when *Helicobacter pylori* is exposed to acid stress (Álvarez and Toledo, 2017). Additionally, they can induce and constrain negative supercoiling in DNA domains and form protein complexes that regulate gene expression or DNA replication (Stojkova et al., 2019). Recently, the participation of HU proteins in mediating cell attachment to extracellular DNA in biofilms was reported (Thakur et al., 2021). Therefore, HU proteins are also likely involved in such conserved essential roles in these model acidophiles.

### 3.4. *Acidithiobacillia* class core NAPs are valuable as single-gene phylogenetic markers

**Figure 4:**
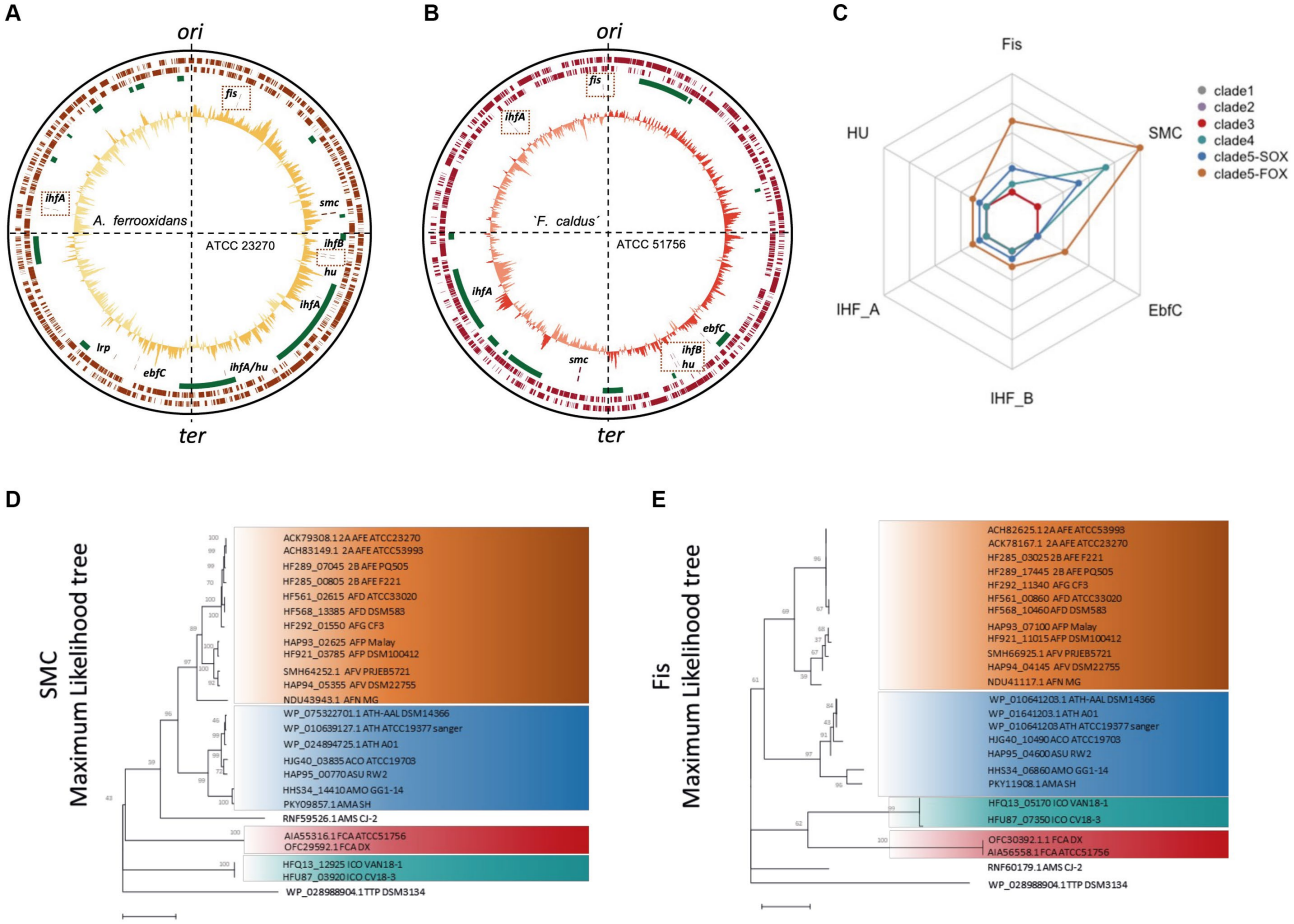
Positional and amino acid sequence conservation of *Acidithiobacillia* class core NAPs. Circular plots of *A. ferrooxidans* ATCC 23270^T^ **(A)** and ‘*F. caldus*’ ATCC 51756^T^ **(B)** chromosomes showing the location of candidate core NAPs. From inner to outer tracks: %GC plot; NAPs location (tan colored lines); and predicted MGEs locations (green boxes). Genomic quadrants with respect to the origin and terminus of replication are indicated in doted lines. **(C)** Average protein dissimilarity (100 – % identity) obtained from pairwise blast comparisons for each core NAP PF among *Acidithiobacilllia* class clades [as defined in Moya-Beltrán et al. (2021)]. Clade notations are as follows: clade 1: TTP, *T. tepidarius* (*n* = 1); clade 2: AMS, ‘*Ambacidithiobacillus sulfuriphilus*’ (*n* = 1); clade 3: ICO, ‘*Igneacidithiobacillus copahuensis*’ (*n* = 6) and IYE, ‘*Igneacidithiobacillus yellowstonensis*’ (*n* = 1); clade 4: FCA, ‘*Fervidacidithiobacillus caldus*’ (*n* = 18); clade 5-SOX: ATH, *A. thiooxidans* (*n* = 20); ACO, *A. concretivorus* (*n* = 1); ASU, *A. sulfurivorans* (*n = 1*); AMA, *A. marinus* (*n* = 1); AMO, *A. monserratensis* (*n* = 1); and clade 5-FOX: AFE, *A. ferrooxidans* (*n* = 13); AFG, *A. ferruginosus* (*n* = 1); AFD, *A. ferridurans* (*n* = 7); AFP, *A. ferriphilus* (*n* = 9); AFV, *A. ferrooxidans* (*n* = 9); AFN, *A. ferrooxidans* (*n* = 1). Maximum-likelihood (ML) phylogenetic trees with bootstrap resampling from alignments of **(D)** SMC (1,000 replicates) and **(E)** Fis (10,000 replicates) protein sequences. Evolutionary history was inferred using ML method by applying Neighbor-Joining and BioNJ algorithms using the JTT model with a discrete Gamma distribution. For SMC and Fis proteins, 10.19 or 13.64% sites were allowed to be evolutionarily invariable, respectively.

Next, we inspected the positional and amino acid sequence conservation of *Acidithiobacillia* class core NAPs. Positional conservation was assessed by comparing two phylogenetically distant *Acidithiobacillia* representatives with closed genomes, *A. ferrooxidans* ATCC 23270^T^ and ‘*F. caldus*’ ATCC 51756^T^ (Figures 4A,B). In both cases, the relative position of core NAP genes with respect to each other was variable, suggesting that significant rearrangements have occurred between genomes of the class during species evolutionary differentiation. In turn, relative position of core NAP-encoding genes with respect to the origin and terminus of replication were conserved. Genes *fis* and *ihfA1* are near the origin of replication, while *ifhB1*, *hup1*, and *efbC* are closer to the replication terminus, albeit at variable locations. The position of the *smc* gene has shifted between these two genomes. This is relevant, since in *E. coli* NAPs expressed preferentially during exponential growth and associated with the higher overall superhelicity are encoded closer to the origin, whereas stationary phase NAPs associated with the lower superhelical density are encoded closer to the replication terminus (Sobetzko et al., 2012). Therefore, it is likely that the conserved relative position of core NAP genes in *Acidithiobacillia* chromosomes (with respect to *E. coli*) is related with both their expression levels at different growth phases, and their role in maintenance and modulation of the nucleoid supercoiling status during growth. Currently, the scarcity of closed genome sequences of representatives for the other *Acidithiobacillia* lineages prevents us from further exploring this aspect for the entire class.

The amino acid sequence variability between pangenome core NAP PFs at the class level was generally small, ranging from 92.5 to 100% of sequence identity (Figure 4C). As a general trend, less variation was observed among deep branching lineages, such as ‘*Igneacidithiobacillus*’ spp. and ‘*Fervidacidithiobacillus*’ spp. compared to the sulfur and ferrous iron oxidizing acidithobacilli, being *A. ferrivorans* the lineage with most divergent core NAPs set (data not shown). Among this set of *core* NAPs, SMC and Fis showed the highest divergence and recapitulated the *Acidithiobacillia* class proposed phylogeny (Figures 4D,E). SMC proteins (1,103 ± 171 amino acids, median of 542 variant positions) showed a higher power to resolve *Acidithiobacillia* lineages than Fis proteins (78.10 ± 5.17 amino acids, median of 27.4 variant positions) due to their larger size and higher number of polymorphic/variant positions. Contrary to Fis, SMC proteins could resolve the *A. ferruginosus* CF3 from the *A. ferrooxidans/A. ferridurans* clade, and also locate *A. ferrianus* as a deep-branching lineage of the ferrous-iron oxidizing *Acidithiobacillia*, resembling the phylogeny obtained by using a ML phylogenetic tree constructed with 107 concatenated single-copy proteins (Moya-Beltrán et al., 2021). Nevertheless, despite its lower resolution power, short-length Fis proteins could be useful as a single-protein phylogenetic marker for taxonomic assignment of low-quality genome assemblies and metagenome assembled genomes (MAGs) lacking the 16S rRNA gene.

### 3.5. Diversification of NAPs within the *Acidithiobacillia* class is due to both gene family expansion and horizontal gene transfer

To explore the underlying causes of the overall increase in the number of NAP PFs observed in candidate ‘*Fervidacidithiobacillus*’ and the “dual physiology” clades of the *Acidithiobacillia* class with respect to deep branching lineages, we focused next on the NAP-encoding genes pertaining to the flexible pangenome. Evidence of possible gene duplication events (gene dose >2) were evident in several species (Supplementary Figure S5), as exemplified by the psychrophilic *A. ferrivorans*. The genome of the type strain (DSM 22755) of this species contained the highest number of genes encoding NAP protein variants, including 8 distinct IHF_A (27.8–94.1% identity), 6 IHF_B (38.4–95.9% identity) and 3 HU (30.2–51.2% identity). Amino acid sequence divergence levels suggest these sets include both paralogs and exogenous NAPs acquired through HGT. Given that the *A. ferrivorans* DSM 22755 has the largest genome analyzed (4.62 Mb), it is tempting to speculate that this large repertoire of flexible NAPs represent an advantageous mechanism to stabilize larger genomes, similar to what has been shown for *Deinococcus radiodurans* during exposure to pH or temperature stress (Nguyen et al., 2009). However, according to the *paralogue index* metric (see methods), true NAPs paralogues seem to be restricted to IHF_A, IHF_B and HU protein families, encoded in the *A. thiooxidans* (2 H-NS and 2 IHF_B paralogues), ‘*A. concretivorus*’ (3 HU paralogues) and ‘*A. sulfurivorans*’ (4 HU and 4 IHF_B paralogues) genomes (Supplementary Figure S5). The fact that overall numbers of candidate HU, IHF_A and IHF_B proteins covary with the number of CONJscan predicted relaxase genes per genome (*r* = 0.701, Supplementary Figure S6A), but not with the number of TnpPred predicted transposases (Riadi et al., 2012) (*r* = 0.083, Supplementary Figure S6B), suggests that many NAPs pertain to transmissible rather than translocative MGEs.

Contrary to what was observed in *core* NAP-encoding genes neighborhoods, analysis of the predicted gene functions encoded in the vicinities of flexible NAPs revealed little conservation. Amongst the most frequent gene products, we consistently noted the presence of proteins related to conjugation (type IV secretion system proteins VirB1, TrbL, TrbJ, and VirD1), chromosome partitioning and plasmid segregation related proteins (ParA and ParM), toxin/antitoxin RelB/RelE systems, integration/recombination related proteins (XerD) and type-I restriction-modification (RM) systems (Table 2 and Supplementary Table S4). Scarce overall sequence conservation, together with a positive association between flexible HU, IHF_A and IHF_B proteins and mobilome signature proteins, represent convincing evidence of *Acidithiobacillia* flexible NAPs affiliation to the episomal and/or integrative mobilome. One clear example of this, is ‘*A. sufurivorans*’ RW2 exclusive NAP ortholog of NdpA/YejK. This candidate *ndpA* gene is located within a gene cluster flanked by integrase/transposase coding genes downstream a tRNA^Met^ and adjacent to the conserved *rpsU-gatB-mutS2-gnaG-rpoD* gene cluster (Supplementary Figure S6C). Neighboring genes to *ndpA* encode a UvrD-helicase domain-containing protein, a site-specific DNA-methyltransferase, an addiction module protein (or toxin-antitoxin system protein) and a DEAD/DEAH box helicase family protein (pseudogene). The presence of transposase/integrase, in addition to an addiction module protein and a DNA-methyltransferase, and two flanking direct sequence repeats, strongly indicated that *ndpA* belongs to an MGE integrated at the tRNA^Met^. NdpA/YejK binding to double-stranded-DNA in *E. coli* nucleoids has been detected in a yeast two-hybrid screen using the ParE subunit of topoisomerase IV as bait (Lee and Marians, 2013). This interaction with the topoisomerase IV can produce a distributive relaxation of negatively supercoiled DNA and stimulate relaxation of positively supercoiled DNA (Lee and Marians, 2013), suggesting that occurrence of this NAP orthologs in strain *A. sulfurivorans* RW2 MGE could play a role in modulating DNA-supercoiling of the MGE during its life cycle.

**Table 2:** Frequent KEGG annotations encoded in *Acidithiobacillia* flexible NAP PFs neighborhoods.

NAP family	KEGG #	KEGG annotation	Frequency (%)^a^
**Alba_2**	K01007	Pyruvate, water dikinase [EC:2.7.9.2]	61.54
	K09803	Uncharacterized protein	53.85
	K00331	NADH-quinone oxidoreductase subunit B [EC:7.1.1.2]	46.15
**H-NS**	K03427	type I restriction enzyme M protein [EC:2.1.1.72]	22.50
	K03469	Ribonuclease HI [EC:3.1.26.4]	17.50
	K07301	Cation:H+ antiporter	15.00
	K09705	Uncharacterized protein	15.00
	K07043	Uncharacterized protein	12.50
	K01153	Type I restriction enzyme, R subunit [EC:3.1.21.3]	10.00
	K01154	Type I restriction enzyme, S subunit [EC:3.1.21.3]	10.00
	K01725	Cyanate lyase [EC:4.2.1.104]	10.00
	K02314	Replicative DNA helicase [EC:3.6.4.12]	10.00
	K12562	Amphiphysin	10.00
	K19302	Undecaprenyl-diphosphatase [EC:3.6.1.27]	10.00
**HU**	K04764	Integration host factor subunit alpha	5.62
	K01602	Ribulose-bisphosphate carboxylase small chain [EC:4.1.1.39]	4.49
	K02448	Nitric oxide reductase NorD protein	4.49
	K03427	Type I restriction enzyme M protein [EC:2.1.1.72]	4.49
	K04748	Nitric oxide reductase NorQ protein	4.49
**IHF_A**	K03194	Type IV secretion system protein VirB1	9.84
	K07344	Type IV secretion system protein TrbL	6.56
	K20266	Type IV secretion system protein TrbJ	6.56
	K07657	OmpR family, phosphate regulon response regulator PhoB	6.01
	K08176	MFS transporter, PHS family, inorganic phosphate transporter	6.01
	K03427	Type I restriction enzyme M protein [EC:2.1.1.72]	5.46
	K03969	Phage shock protein A	5.46
	K04763	Integrase/recombinase XerD	4.92
	K04764	Integration host factor subunit alpha	4.92
	K18640	Plasmid segregation protein ParM	4.92
	K01992	ABC-2 type transport system permease protein	4.37
	K03496	Chromosome partitioning protein ParA	7.89
	K01153	Type I restriction enzyme, R subunit [EC:3.1.21.3]	6.58
	K03205	Type IV secretion system protein VirD4	6.58
	K07283	Putative salt-induced outer membrane protein	6.58
**KfrA**	K03496	Chromosome partitioning protein ParA	34.21
	K06218	mRNA interferase RelE/StbE	13.16
	K18918	Antitoxin RelB	13.16
	K21528;K14060	Serine recombinase [EC:3.1.22.- 6.5.1.-]	10.53

^a^Frequency percentages correspond to the KEGG-based vicinity frequency index (see methods).

### 3.6. DNA-bending NAPs occur in episomal mobile genetic elements of the class

To further characterize the *Acidithiobacillia* NAP PFs belonging to the flexible gene compartment, we analyzed candidate NAPs encoded in known plasmids of the class. Analysis of 26 publicly available *Acidithiobacillia* plasmid sequences allowed us to unambiguously link 14 candidate NAP-encoding gene orthologs to 7 different plasmids (Table 3 and Supplementary Table S5). As a general trend, we found that small-sized plasmids such as *A. ferrivorans* PQ33 pAfPQ33_1 (10.2 kb) or ‘*F. caldus*’ SM1 pLAtc2 (14.1 kb) harbored only one NAP, medium-sized plasmids such as pAca1.1 (27.3 kb) and p2 (32.8 kb) in ‘*F. caldus*’ ATCC 51756 and MTH-04, respectively, encoded two NAPs, and megaplasmids also occurring in ‘*F. caldus*’ such as the MTH-04 p1 (190 kb) encoded up to 4 NAPs per replicon. Previous reports have suggested that plasmid-encoded NAPs may contribute to host cell fitness and play a role in maintenance of larger plasmids (Takeda et al., 2011). Presently, it is impossible to ascertain if this direct relationship between plasmid size and NAPs numbers extends to all *Acidithiobacillia* linages, since plasmids data in several of them is scarce or missing.

**Table 3:** Plasmid-encoded candidate NAPs identified in *Acidithiobacillia* class genomes.

Specie/strain^#^	Plasmid	Size (kb)	CDS	NAPs number	NAP families	Protein ID	Accession ID
*A. ferrivorans* PQ33	pAfPQ33_1	10.2	10	1	KfrA	ARU59618.1	CP021414.1
*A. ferrivorans* CF27	AFERRIp	46.4	50	1	KfrA	SMH67819.1	LT841306.1
*`F. caldus * ATCC 51756	pACA1.1	27.5	33	2	KfrA; IHF_A	AIA56783.1; AIA56791.1	CP005988.1
*`F. caldus * MTH-04	p1	190.8	229	4	HU; HU; IHF_A; HU	AUW34137.1; AUW34251.1; AUW34139.1; AUW34211.1	CP026329.1
*`F. caldus * MTH-04	p2	32.8	66	2	IHF_A; KfrA	AUW34268.1; AUW34286.1	CP026330.1
*`F. caldus * SM-1	mega plasmid	251.8	255	2	HU; IHF_A	AEK59566.1; AEK59568.1	CP002574.1
*`F. caldus * SM-1	pLAtc3	29.7	28	1	IHF_A	AEK59821.1	CP002577.1
*`F. caldus * SM-1	pLAtc2	14.1	14	1	IHF_A	AEK59799.1	CP002576.1

^#^Species names as proposed in Moya-Beltrán et al. (2021).

Candidate NAPs encoded in *Acidithiobacillia* plasmids belonged to the IHF_A (*n* = 5), HU (*n* = 4) and KfrA (*n* = 5) protein families, and were exclusively found in moderate thermophilic *`F. caldus * (12 NAPs) and psychrophilic *A. ferrivorans* (2 NAPs) strains, two species with acknowledged presence of plasmids (Rawlings, 2005; Acuña et al., 2013; Tran et al., 2017). Potential roles for plasmid-encoded NAPs, include plasmid replication, maintenance, transfer and integration into host cells chromosomes (Takeda et al., 2011; Shintani et al., 2015). These roles are likely conserved in plasmid-encoded NAPs of the *Acidithiobacillia* class. Yet, apart from KfrA, the only NAP families detected in plasmids of the class, were HU, IHF_A and IHF_B, all of which are well known for their role in DNA bending. In this regard, the presence of IHF-binding sites in the *ori* region of several *A. ferrooxidans* pTF5-like plasmids is noteworthy (Chakravarty et al., 1995; Moya-Beltrán et al., 2023). These findings suggest that plasmid-encoded NAPs of the acidithiobacilli may participate in NAP induced DNA-bending within the *ori* and/or *oriT* regions to facilitate replication and/or transfer processes, as is the case for IHF proteins during *E. coli* pSC101 plasmid replication (Biek and Cohen, 1989) and IncFV plasmid pED208 DNA-transfer initiation (Di Laurenzio et al., 1995).

### 3.7. Genes encoding for DNA-bending NAPs occur in large integrated mobile genetic elements of the class

We also assessed the presence of candidate NAP-encoding genes in known *Acidithiobacillia* integrated MGEs (iMGEs) identified previously in strains with closed genomes (Supplementary Table S6). Overall, 13 candidate NAPs were linked to 8 different mobile elements, in 6 different strains. NAP-encoding genes ranged from 1 to 3 per element, and invariantly pertained to IHF_A, IHF_B, HU and Alba_2 families (Table 4). All NAP-encoding iMGEs were Integrative Conjugative Elements (ICE), and ranked among largest elements in each genome analyzed (>80 kb). Among these, were the active-excising elements of *A. ferrooxidans* ATCC2320 (ICE*Afe*1 and ICE*Afe*2) and ‘*F. caldus*’ strains ATCC 51756 and SM-1 (ICE*Aca*TY.2 and ICE*Aca*SM.2) containing functional Tra-and Trb-type T4SS, for which the presence of both integrated and excised forms of the elements has been demonstrated (Bustamante et al., 2012; Acuña et al., 2013; Flores-Ríos et al., 2019a). The *A. ferrooxidans* CCM4253 genome showed the highest amount of integrated MGE-encoded NAPs, with 7 predicted NAPs distributed in 3 different MGEs. All identified NAP-encoding genes mapped in the vicinity of conjugative, integration/excision and restriction/modification functions (see details in Supplementary Table S6). Based on their ubiquity and location, these MGE-encoded DNA-bending NAPs are likely to aid in ICE integration into the host chromosome, or either during the elements excision, as has been previously shown in other bacteria (e.g., uropathogenic *E. coli* strain 536, Chittò et al., 2020). Another plausible scenario is that these proteins assist T4SS-mediated DNA-transfer initiation of the conspicuous ICE elements (Di Laurenzio et al., 1995), like those found in the acidithiobacilli. Regardless of the mechanism, the presence of *ihfA*, *ihfB* and *hupA* exclusively in conjugative elements point to an important contribution of these proteins to ICE molecular biology in these acidophiles.

**Table 4:** MGE-encoded candidate NAPs identified in *Acidithiobacillia* class genomes.

Species/strain^#^	MGE	Size (kb)	CDSs	NAP families	Protein_id	Relevant genetic context
*A. ferrooxidans* ATCC 23270	ICE*Afe*1	291.3	364	IHF_A	ACK80120.1	TraFHG
*A. ferrooxidans* ATCC 23270	ICE*Afe*2	166.1	197	IHF_A; HU	ACK79793.1; ACK80433.1	Type-I RM system
*A. ferrooxidans* CCM 4253	ICE iMGE4	136.0	144	HU; IHF_A	PZD81251.1; PZD81252.1	Type-I RM system
*A. ferrooxidans* CCM 4253	ICE iMGE10	81.0	95	IHF_A; IHF_B	PZD82438.1; PZD82449.1	Type-I RM system/site-specific integrase
*A. ferrooxidans* CCM 4253	ICE iMGE13	115.0	125	IHF_A; HU; IHF_A	PZD81822.1; PZD81833.1; PZD81875.1	EAL/bcs operon /relaxase-mobilization nuclease
‘*F. caldus*’ ATCC 51756	ICE*Aca*_TY.2_	183.3	183	IHF_A	AIA55711.1	type-I RM system/site-specific integrase
‘*F. caldus*’ SM-1	ICE*Aca*_SM.2_	208.3	202	HU	AEK57916.1	
*A. ferrooxidans* ATCC 53993	ICE iMGE6	99.6	102	Alba_2	ACH84262.1	Type-III RM *res* subunit; *brnTA* addiction module; *mobM* relaxase.

^#^Species names as proposed in Moya-Beltrán et al. (2021); ICE names as proposed in Bustamante et al. (2012), Acuña et al. (2013), and Moya-Beltrán et al. (2023).

**Figure 5:**
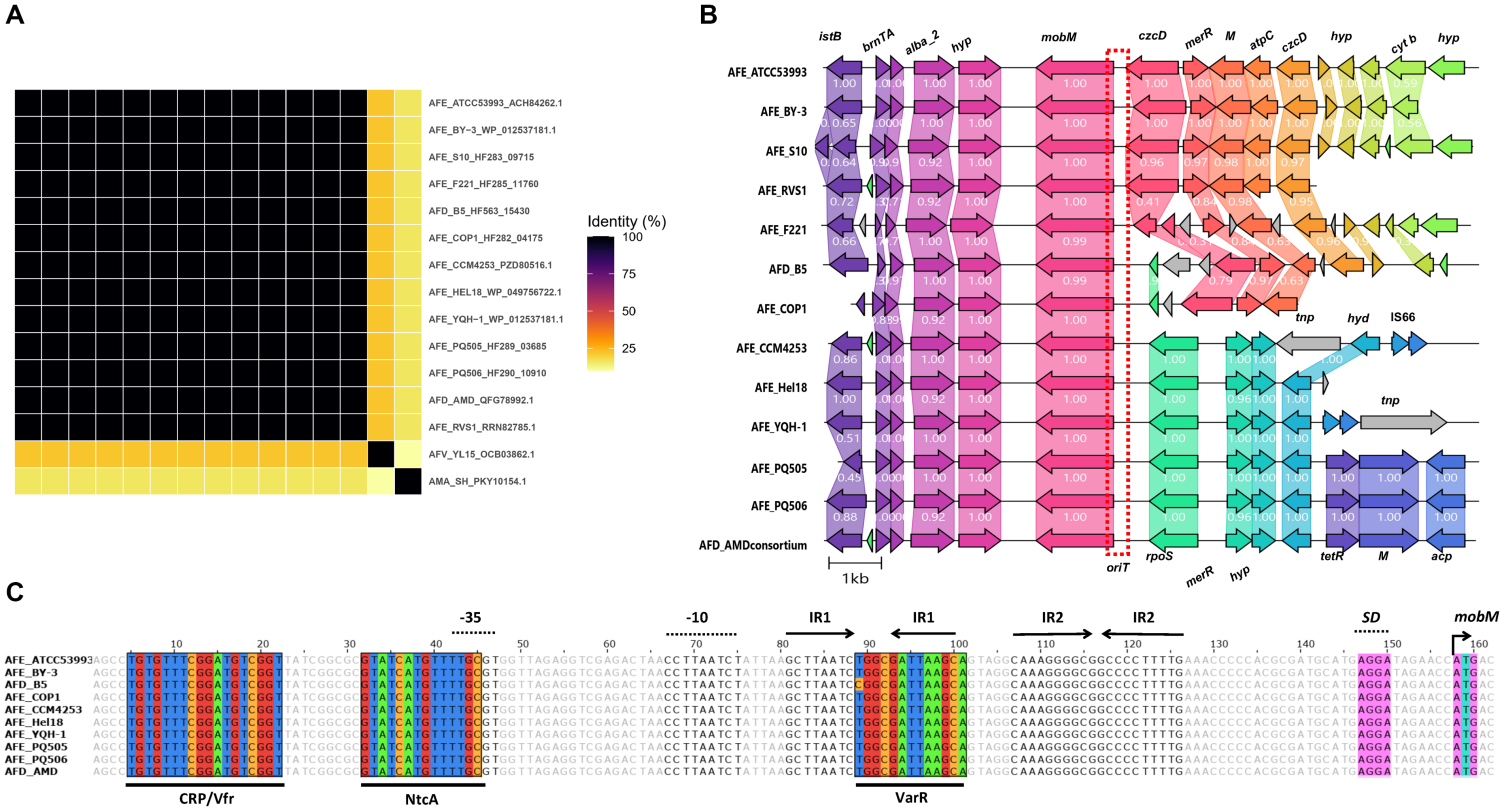
Conserved AlbA_2 NAP PF in iMGEs of *Acidithiobacillia* class representatives. **(A)** Clustering analysis of Alba_2 proteins based on pairwise BlastP identity values, shown as heatmap. **(B)** Gene vicinities of candidate Alba_2 NAPs in iron oxidizing *A. ferrooxidans, A. ferridurans* and *A. ferrivorans* strains. Conserved occurrence of a MobM relaxase and toxin-antitoxin system genes (*brnTA*) in the immediacy of *albA*_*2* suggest iMGE6 is an Integrative Mobilizable Element (IME). Only a representative segment of each contig (up to 10 kb upstream and downstream Alba_2 CDS) is depicted. **(C)** Putative *oriT* region upstream the *mobM* gene, containing two sets of inverted repeats sequences (IR1 and IR2) and putative binding sites corresponding to Prodoric matrix CRP/Vfr (MX000371 and MX000110, scores of 6.22 and 6.91 respectively), NtcA (MX000209, score of 9.69) and VarR (MX000401, score of 6.03) are shown. Predicted-35 and-10 regions for sigma 70 promoter and SD, Shine-Dalgarno sequence are shown upstream of putative *mobM* initiation codon. Gene names abbreviations used are: *res*, type-III restriction protein *res* subunit; *GNAT*, GNAT family *N*-acetyltransferase; *nuoB,* NADH-quinone oxidoreductase subunit B*; pep*, phosphoenolpyruvate-utilizing protein mobile región; *istB,* IstB domain ATP-binding protein; *copG,* CopG DNA-binding domain; *alba_2*, Alba_2 DNA-binding domain containing protein; *hyp,* conserved hypothetical protein; *mobM,* MobM relaxase family protein; *czcD*, Co/Zn/Cd efflux system component; *merR*, Pb/Cd responsive transcriptional regulator MerR family; M, methyltransferase; *atpC*, ATP synthase F1, epsilon subunit; *cyt b,* cytochrome *b*/*b*6 containing protein, nickel-dependent hydrogenases *b*-type cytochrome subunit; *rpoS*, RNA polymerase sigma-54 factor; *tnp*, ISL3-like element ISTfe1 or IS66-like element ISAfe4 family transposase; *hyd*, alpha/beta hydrolase; IS66, IS66 family insertion sequence hypothetical protein; M, methyltransferase; *acp*, beta-ketoacyl-ACP synthase II. Red dotted box shows a putative *oriT* region upstream *mobM* coding gene.

A set of 13 highly conserved AlbA_2 protein encoding genes (Figure 5A) were found exclusively in ferrous-iron oxidizing *A. ferrooxidans, A. ferridurans* and *A. ferrivorans* strains (excepting ‘*A. marinus*’ SH which encoded a highly diverged ortholog), mapping in a recently acknowledged iMGE of *A. ferrooxidans* strains (genomic island ATCC53993_iMGE6, Moya-Beltrán et al., 2023). The immediate gene vicinity of candidate *albA_2* genes is also highly conserved, encoding a type II toxin-antitoxin system upstream (BrnA, BrnT) and a MobM-family relaxase family protein downstream (Figure 5B). Inspection of the nucleotide sequence upstream putative *mobM* initiation codon showed a putative *oriT* region including two sets of characteristic inverted repeat regions, with 8 and 10 nucleotides-long stems respectively, and a 4 nucleotides-long loops (Figure 5C). This structure resembles the replication origin of streptococcal plasmid pMV158, where a recognizable site for a nicking enzyme that cuts one DNA strand - as required for mobilization of the plasmid (*nic* site) - is located in the loop of the IR1 site (Francia et al., 2004). In addition to *albA2* orthologs, the elements identified in these iron-oxidizing acidithiobacilli also bared the above reconned HU and IHF DNA bending NAPs, either as single genes (*hup*) or as contiguous triplets (*ihfA*-*hup*-*ihfA*). Although the iMGE6-like elements have been described as integrated genomic islands, the presence of the mobilization hallmark genes prompt their reclassification as Integrative Mobilizable Elements (IMEs). Although AlbA_2 domain containing proteins are known to be widely distributed in archaea, bacteria and a number of eukaryotes, its association with bacterial MGEs had not been previously established. Further research is needed to understand the potential functions and implications of the AlbA_2 NAPs in MGEs biology.

### 3.8. Flexible NAPs are useful signatures to identify MGEs in *Acidithiobacillia* genome sequences

**Figure 6:**
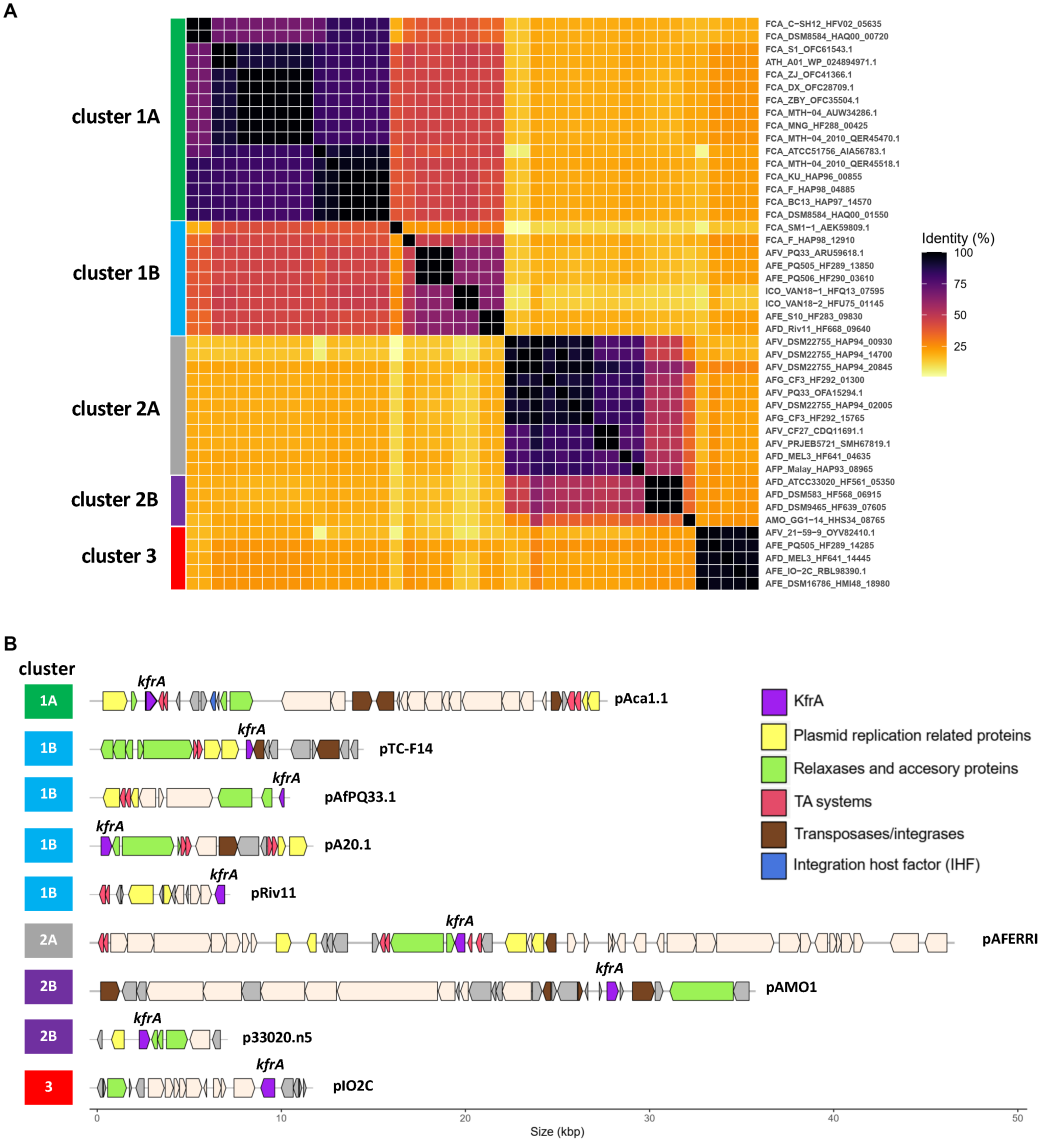
KfrA proteins in *Acidithiobacillia* class plasmids. **(A)** Clustering analysis of KfrA proteins based on pairwise BlastP identity values, shown as heatmap. Five main clusters and a number of subclusters were recognized. **(B)** Representative plasmids and plasmid-like contigs containing genes encoding KfrA protein variants in selected *Acidithiobacillia.* Known plasmids were chosen as representatives of KfrA clusters 1A (pAca1.1), 1B (pTC-F14), 1B (pAfPQ33) and 2A (pAFERRI). Additional clusters grouped exclusively novel candidate plasmid-like elements: 1B (pA20.1), 1B (pRiv11.1), 2B (pAMO.1), 2B (p33020.n5), and 3 (pIO2C). KfrA DNA-binding protein coding genes (purple) are indicated. Predicted plasmid replication proteins (yellow), MOB relaxases (green), toxin-antitoxin systems (red), transposases/integrases (brown) and integration host factor alpha subunits (blue) coding genes are also indicated.

Given the ubiquity of flexible NAP-encoding genes in episomal and integrated MGEs, we next assessed the value of *Acidithiobacillia* flexible NAPs as seed sequences to identify novel MGEs. To this end, we focused on 2 frequently occurring flexible NAPs, KfrA and H-NS. Using this strategy, various as-of-yet unknown episomal and integrated MGEs or fragments of these, were uncovered from both complete and draft genomes of the class. We first inspected genomic loci and unassembled contigs gathered using the plasmid-related protein KfrA. Clustering analysis of KfrA family orthologues revealed 5 main amino acid sequence variants of the protein, named clusters 1A, 1B, 2A, 2B, and 3 (Figure 6A). KfrA-cluster 1 and cluster 2 held the largest number of hits and matches to already known plasmids of ‘*F. caldus*’ and *A. ferrivorans* strains (Figure 6B), represented by the ‘*F. caldus*’ type strains pAca1.1 plasmid (cluster 1A, Acuña et al., 2013) and the *A. ferrivorans* CF27/PRJEB5721 pAFERRI plasmid (cluster 2A, Tran et al., 2017). Undescribed plasmid candidates were also retrieved for *A. thiooxidans,* ‘*A. ferruginosus*’ and *A. ferriphilus* less-well characterized strains. KfrA-cluster 1B variant mapped to the pTC-F14 ‘*F. caldus*’ plasmid (Rawlings, 2005) and pAfPQ33-plasmid from *A. ferrivorans* (Ccorahua-Santo et al., 2017), and two other strains isolated from Pasco, Perú. Additional cluster 2B and 3 variants harbored mostly novel plasmid candidates pertaining to 4 *A. ferridurans*, 3 *A. ferrooxidans*, 1 *A. ferrivorans* and 1 ‘*A. monserratensis*’ strain. These plasmid-like contigs also bared genes encoding plasmid replication (*repABC*), mobilization (*mobABCDE*), segregation (*parAG*), toxin/antitoxin stabilization systems, along with plasmid cargo genes, such as c-di-GMP EAL/PilZ-containing control module (Figure 6B). Presence of these plasmid hallmark genes, related to key aspects of plasmid biology, strongly suggest that the identified contigs correspond to novel plasmids of the class. Interestingly, most of *ihfA* (8 out of 9 proteins) and *ihfB* (5 out of 6) found in these plasmid-like contigs encoded proteins belonging to a single IHF_A or IHF_B PF, suggesting the coevolution of these NAPs within the plasmid-like contigs, behaving as cohesive heritable units.

We next focused on the conservation and location of *Acidithiobacillia* of H-NS encoding gene orthologs, a major component of the *E. coli* nucleoid. In *E. coli* H-NS acts as a global transcriptional regulator, modulating both transcription initiation and elongation stages (Grainger, 2016), while in *Salmonella* H-NS binding preferences for AT-rich regions has prompted the recognition of its role in foreign-DNA silencing (Navarre et al., 2007). H-NS orthologs were identified exclusively in strains of the “dual physiology” (iron/sulfur) clade of *Acidithiobacillia*, yet not in all its acknowledged species (Moya-Beltrán et al., 2021). Due to this scattered pattern of occurrence, it is unlikely that H-NS plays the essential role that it does in *E. coli*, and it is more likely that it forms part of the mobilome of this restricted group of species (Figure 2C). *Acidithiobacillia* class H-NS orthologues showed lower heterogenicity at the amino acid-sequence level, clustering as 3 variants with unbalanced representativity in the analyzed set: (a) cluster-1 variants occurring principally in *A. thiooxidans* strains [*n* = 37, 85.2–100% identity], (b) cluster-2 variants occurring almost exclusively in *A. ferrivorans* strains [*n* = 7, 92.7–100% identity] and (c) cluster-3 variant, present only in *A. ferriphilus* ST2 (Supplementary Figure S7A). Analysis of H-NS variants immediate genes contexts revealed subgroups with partially conserved MGE-like features (Supplementary Figure S7B), including type-I restriction-modification systems, unclassified methyltransferase and endonuclease coding genes, toxin/antitoxin systems, recombinases/integrases, and many hypotheticals. Both the *hns* gene location and its pattern of occurrence, restricted to the two species with the larger genomes in the class (*A. thiooxidans* and *A. ferrivorans*), suggests H-NS could act as xenogeneic silencer favoring the acquisition and integration of DNA from exogenous sources into these strains chromosomes, while avoiding the burden of unregulated expression of newly acquired genes.

To test this hypothesis we analyzed RNA-seq data obtained from *A. thiooxidans* ATCC 19377 cells grown on elemental sulfur, and inspected the expression patterns of the genes in the vicinity of *hns*. Results obtained showed that the H-NS coding gene is located within a predicted ICE spanning 152 kb. The H-NS coding gene, as well as other genes located in its immediate vicinity, including a DNA polymerase IV (*dinB*), an error-prone DNA polymerase (*umuD*) and a toxin/antitoxin system (*TA*), were expressed at low levels, approaching the genome average (RNA-seq coverage ~1.0). However, the vast majority of the regions within the predicted MGE showed negligible transcription levels (Supplementary Figure S8). Although H-NS mediated xenogeneic silencing of this MGE serves as a plausible explanation for these results, demonstration of causality requires further experimental work.

## 4. Conclusion

By using a combination of comparative genomic and phylogenetic strategies we analyzed 93 genomes of the different species that conform the *Acidithiobacillia* class and identified a total of 1,197 NAPs belonging to 12 different protein families. Pangenome analyses showed a conserved signature of 6 NAPs encoded in all *Acidithiobacillia* class sequenced species, most likely representing an essential set of housekeeping proteins needed for chromosome condensation, maintenance, and partitioning, along with other DNA transactions. This set of single-copy genes could be useful as potential chromosome-architecture structural markers, and as completeness assessment markers for metagenome-assembled and/or draft genomes. Phylogenetic analysis of core NAPs showed that both SMC and Fis proteins sensibly and accurately recapitulate the proposed phylogeny of the class, a much-sought property in discriminant markers. A positive correlation between the number of flexible NAPs and genome size was uncovered in this work, which is largely explained by an increase in the number of HU, IHF_A and IHF_B variants in species with larger genomes. Thus, expansion and/or acquisition of NAP coding genes closely accompanies species diversification in this taxon. Frequent association of *Acidithiobacillia* class flexible NAPs with both episomal and integrated MGEs, offer additional causalities to this observation. MGE-associated NAPs are likely to play diverse roles including: (i) aiding in replication and/or maintenance of episomal MGEs, (ii) mediating integration/excision of episomal/integrated MGEs, (iii) modulating transfer of mobilizable elements, and/or (iv) silencing expression of MGE-encoded genes. Hypothesis emerging from this analysis will surely guide future experimental research and help determine the specific roles each NAP PFs plays in *Acidithiobacillia* class physiology and adaptation to their harsh environments.

## Data availability statement

The original contributions presented in the study are included in the article/supplementary material, further inquiries can be directed to the corresponding author.

## Author contributions

SB: Data curation, Formal analysis, Investigation, Methodology, Validation, Visualization, Writing – original draft, Writing- review & editing. AM-B: Visualization, Writing – review & editing, Data curation, Formal analysis, Methodology, Software. DS-G: Methodology, Software, Writing – review & editing. CV: Data curation, Formal analysis, Writing – review & editing. TP-A: Conceptualization, Resources, Supervision, Writing – review & editing. AL: Conceptualization, Resources, Supervision, Writing – review & editing. RQ: Conceptualization, Formal analysis, Funding acquisition, Investigation, Resources, Supervision, Writing – original draft, Writing – review & editing. SB: Writing- review & editing.

## Funding

The author(s) declare financial support was received for the research, authorship, and/or publication of this article. This work was supported by grants from Agencia Nacional de Investigación y Desarrollo (ANID) FONDECYT 1221035 (RQ), FONDECYT 1200577 (AL), FONDECYT 1211045 (TP-A), Exploración 13220230 (SB and RQ), and Financiamiento Basal para Centros Científicos y Tecnológicos de Excelencia de ANID AFB 210008 (RQ, AL, and TP-A), Centro Ciencia & Vida PostDoc HINGE (SB), and Vicerrectoría de Investigación y Doctorados Universidad San Sebastián USS-FIN-23-PDOC-03 (AM-B). This publication received support from the Vicerrectoría de Investigación y Doctorados de la Universidad San Sebastián – Fondo VRID_APC23/17.

## Conflict of interest

The authors declare that the research was conducted in the absence of any commercial or financial relationships that could be construed as a potential conflict of interest.

The author(s) declared that they were an editorial board member of Frontiers, at the time of submission. This had no impact on the peer review process and the final decision.

## Publisher’s note

All claims expressed in this article are solely those of the authors and do not necessarily represent those of their affiliated organizations, or those of the publisher, the editors and the reviewers. Any product that may be evaluated in this article, or claim that may be made by its manufacturer, is not guaranteed or endorsed by the publisher.

## Footnotes

^1^ https://www.ebi.ac.uk/Tools/seqstats/emboss_pepstats/

^2^ https://github.com/wilkox/gggenes

^3^ http://www.softberry.com/

^4^ https://www.prodoric.de/vfp/

^5^ http://tree.bio.ed.ac.uk

^6^ https://www.ncbi.nlm.nih.gov/sra
