# Supplementary material for: Pangenome-level analysis of nucleoid-associated proteins in the Acidithiobacillia class: insights into their functional roles in mobile genetic elements biology
Source: Front Microbiol. 2023 Sep 25;14:1271138. doi: 10.3389/fmicb.2023.1271138 (PMC10561277; doi:10.3389/fmicb.2023.1271138)
Supplement: Supplementary file 7 [file Data_Sheet_1.PDF]

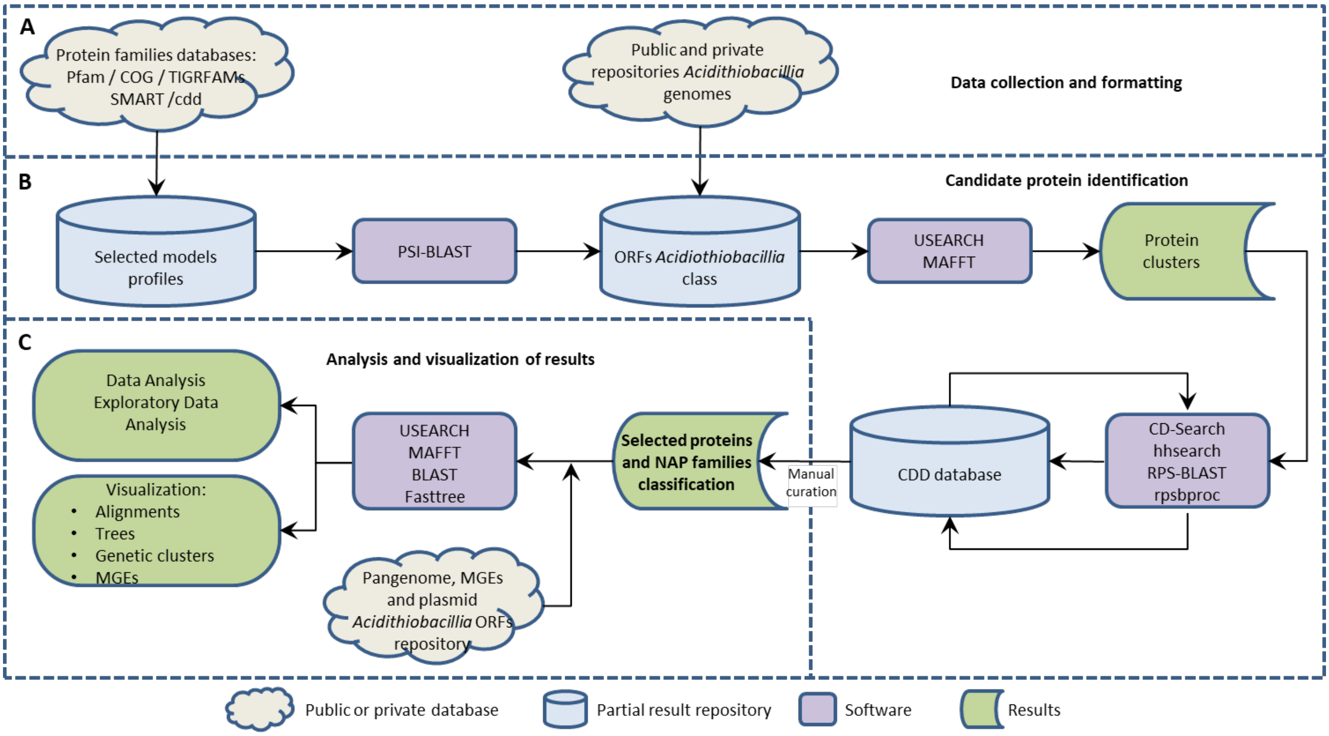

Supplementary Figure 1. *In silico* analysis workflow. Overview of the datasets and general strategy used in the study. (**A**) Data collection and formatting. (**B**) Candidate protein identification. (**C**) Analysis and visualization of results. Symbols are shown in the bottom menu. This figure was adapted from Moya-Beltrán *et al.* 2019. doi:10.3389/fmicb.2019.00381)
