# Supplementary material for: Pangenome-level analysis of nucleoid-associated proteins in the Acidithiobacillia class: insights into their functional roles in mobile genetic elements biology
Source: Front Microbiol. 2023 Sep 25;14:1271138. doi: 10.3389/fmicb.2023.1271138 (PMC10561277; doi:10.3389/fmicb.2023.1271138)
Supplement: Supplementary file 8 [file Data_Sheet_2.PDF]

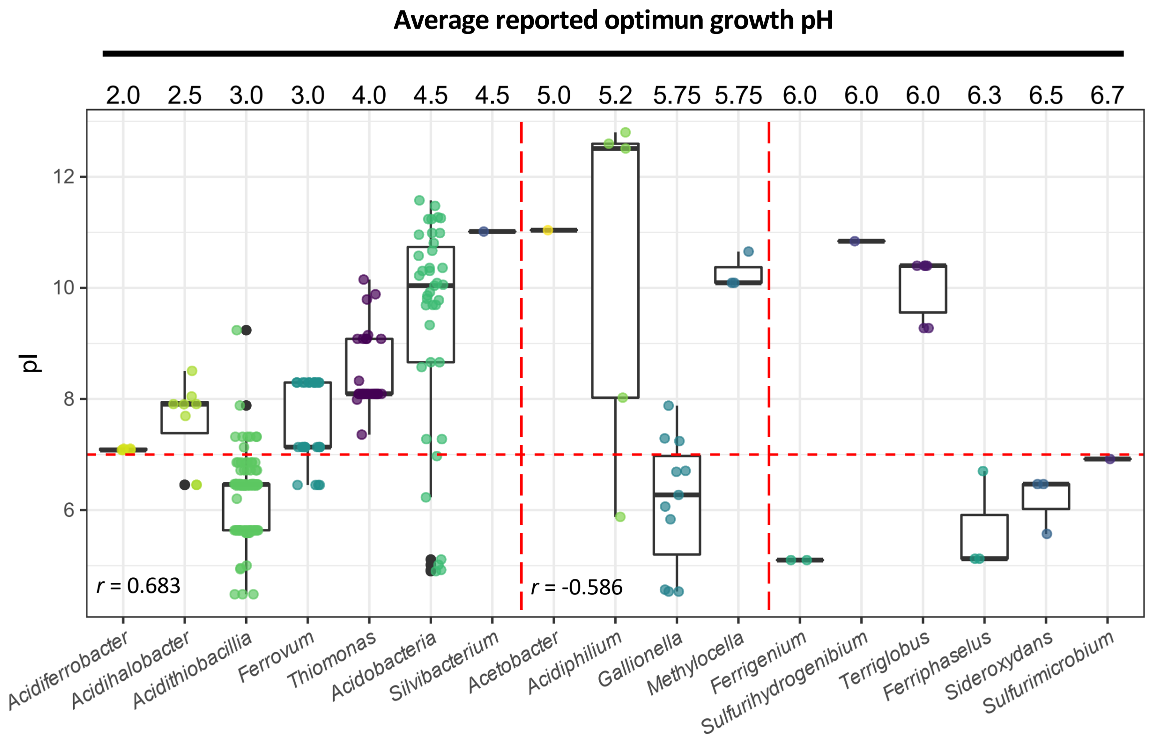

Supplementary Figure 2. Distribution of Fis protein predicted isoelectric points (pI) in "true acidophiles" (optimal growth pH < 3.0), moderate acidophile (optimal growth between pH 3.5 and 4.5) and acid tolerant genera (optimal growth below pH<6). The average optimal pH reported for each taxonomic group is shown at the top. pI predictions were done with Pepstats (https://www.ebi.ac.uk/Tools/seqstats/emboss_pepstats/). Pearson's correlation coefficient (*r*) between the median pI of the predicted Fis proteins and the optimum pH of growth among the extreme and moderate acidophiles (optimal growth pH < 4.5, *r* = 0.683) and among mild acid tolerance genera (optimal growth pH > 4.5 and < 6.0, *r* = -0.586) are indicated. Protein IDs for Fis from acidophiles included in the analyses are listed bellow. *Acetobacter* (OUI99845.1), *Acidiferrobacter* (RCN58303.1, WP_110135648.1, AWP22013.1, WP_065969021.1, OCX46121.1), *Acidihalobacter* (WP_197495992.1, AOU99486.1, WP_083250585.1, WP_076837670.1, WP_038092431.1, APZ44047.1, AOV18385.1, OBS09648.1), *Acidiphilium* (WP_148361035.1, OYW06336.1, OYV81853.1, OYV66252.1, GAN73648.1), *Acidobacteria* (KAA6459659.1, TDI13368.1, RPJ81065.1, RPI23367.1, RMG52487.1, PYX62623.1, PYX53488.1, PYX49871.1, PYX33049.1, PYX25302.1, PYX15988.1, PYV40328.1, PYV04822.1, PYV00779.1, PYU95447.1, PYU70370.1, PYU60302.1, PYU40402.1, PYU29707.1, PYU03856.1, PYU00844.1, PYT90260.1, PYT81947.1, PYT72166.1, PYT65381.1, PYT55290.1, PYT37448.1, PYS74800.1, PYS74631.1, PYS38051.1, PYS21751.1, PYR27623.1, PYR21801.1, PYQ25529.1, PWT80309.1, PIE91065.1, PIE02849.1, OYW05735.1, OLE87253.1), *Ferrigenium* (WP_212785775.1, BBJ00549.1), *Ferriphaselus* (BBE49884.1, WP_062625667.1, WP_051937760.1), *Ferrovum* (QWY76370.1, QWY73616.1, QKE38433.1, QKE40966.1, WP_056929038.1, OZB31914.1, OYV94775.1, OYV80460.1, WP_067495277.1, WP_031597880.1, KXW58516.1, KXW55532.1, KRH79169.1, QWY76370.1, QWY73616.1, QKE38433.1, QKE40966.1, WP_056929038.1, OZB31914.1, OYV94775.1, OYV80460.1, WP_067495277.1, WP_031597880.1, KXW58516.1, KXW55532.1, KRH79169.1), *Gallionella* (KAF0205688.1, ADL56657.1, TXT26925.1, TNC96816.1, TAN75062.1, TAJ79903.1, PIY05602.1, PIV47974.1, OIO82532.1, OIO77028.1, WP_013294576.1), *Methylocella* (WP_166796054.1, VTZ28144.1, VFU16383.1), *Sideroxydans* (ADE10534.1, OHC89087.1, WP_013028433.1), *Silvibacterium* (WP_050058498.1), *Sulfurihydrogenibium* (PMP63050.1), *Sulfurimicrobium* (BCB28678.1), *Terriglobus* (WP_047488859.1, WP_083345275.1, WP_041592662.1, WP_047488859.1, WP_083345275.1, WP_041592662.1) and *Thiomonas* (CQR39617.1, CQR37982.1, CQR39812.1, VDY17934.1, VDY12859.1, VDY08222.1, VDY04606.1, WP_112485148.1, WP_055449190.1, OZB70117.1, OZB69922.1, OZB58587.1, OZB43638.1, OZB43311.1, OYV36165.1, OYV31609.1, WP_094160550.1, SCC92078.1, SBP86664.1, SBP87639.1, WP_079418276.1, ODU96075.1, CQR43368.1, CQR43895.1, CQR38947.1, CUA93460.1, CDW94040.1, CAZ89857.1, WP_031407069.1, WP_013107110.1, WP_018914995.1). *Acidithiobacillia* accession IDs are listed in Supplementary Table 1.
