# Supplementary material for: Pangenome-level analysis of nucleoid-associated proteins in the Acidithiobacillia class: insights into their functional roles in mobile genetic elements biology
Source: Front Microbiol. 2023 Sep 25;14:1271138. doi: 10.3389/fmicb.2023.1271138 (PMC10561277; doi:10.3389/fmicb.2023.1271138)
Supplement: Supplementary file 9 [file Data_Sheet_3.PDF]

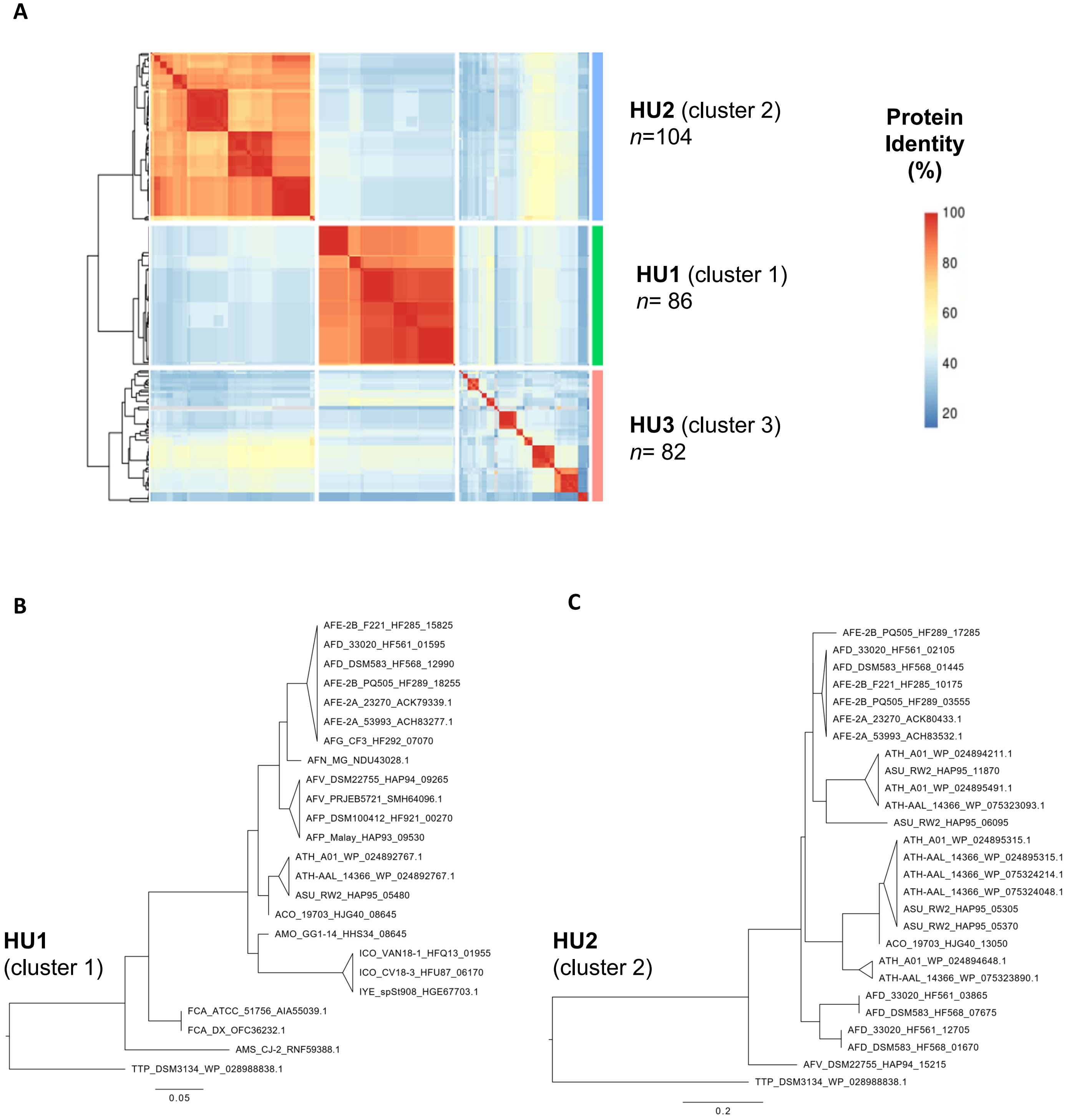

Supplementary Figure 3. NAPs of the HU protein family recovered from *Acidithiobacillia* class genomes. **(A)** Clustering analysis of HU proteins based on pairwise blast identity percentages, shown as heatmap. Proteins were clustered based on their reciprocal BlastP identity values and colored according to the color bar in the figure margin. Clusters are numbered in correlative order according to the relative abundances of their members as HU1 (cluster 1), HU2 (cluster 2) and HU3 (cluster 3). Maximum-likelihood phylogenetic trees from aminoacidic sequence alignments of (**B**) HU1 protein variants (cluster 1) or (**C**) HU2 protein variants (cluster 2). The HU protein of *Thermithiobacillus tepidarius* was included as outgroup. Due to the variability of the HU3 proteins (cluster 3), and the branching pattern inconsistent with the phylogeny of the class (not shown), these were classified as part of the flexible (non conserved) gene complement.
