# Supplementary material for: Pangenome-level analysis of nucleoid-associated proteins in the Acidithiobacillia class: insights into their functional roles in mobile genetic elements biology
Source: Front Microbiol. 2023 Sep 25;14:1271138. doi: 10.3389/fmicb.2023.1271138 (PMC10561277; doi:10.3389/fmicb.2023.1271138)
Supplement: Supplementary file 10 [file Data_Sheet_4.PDF]

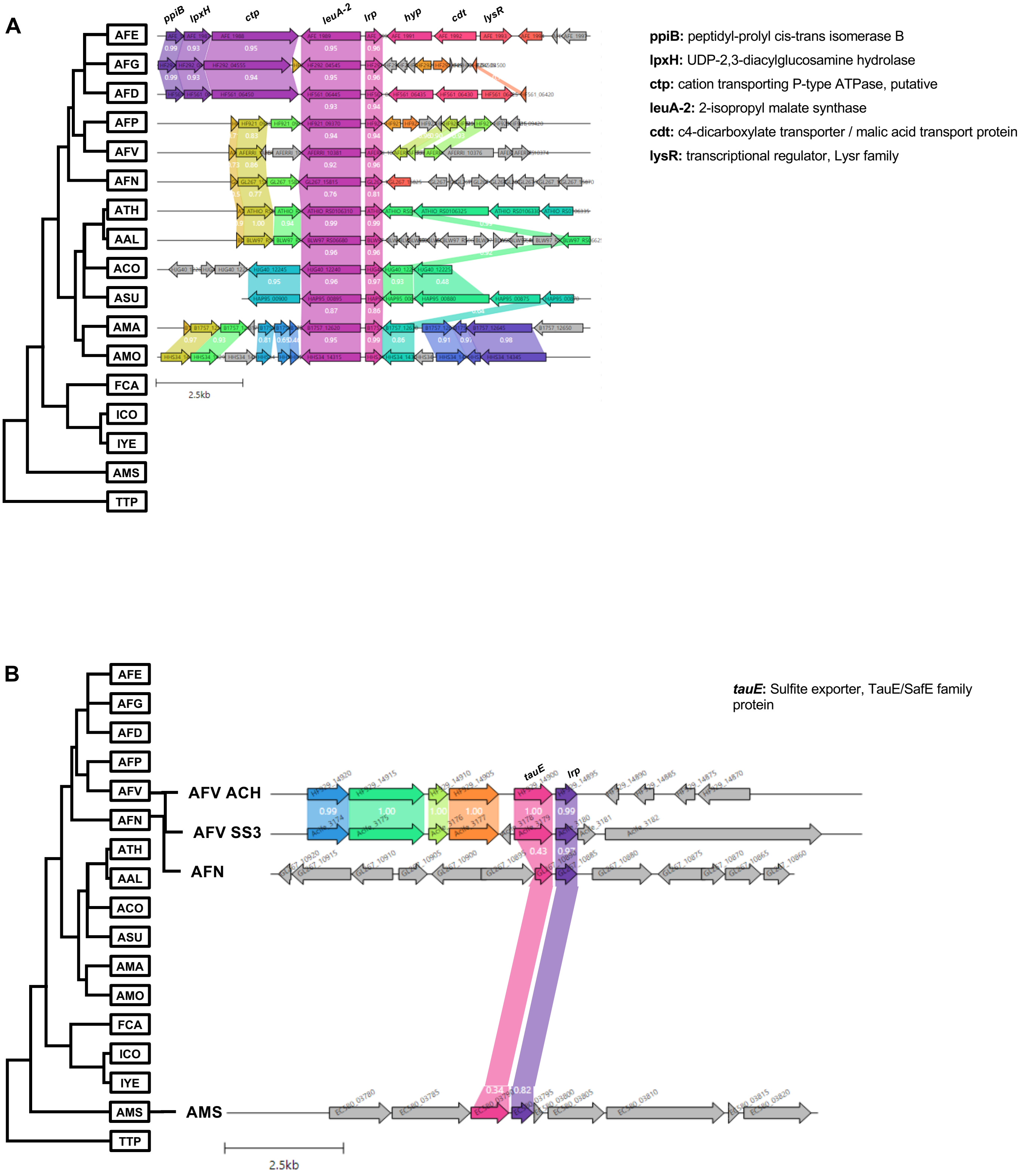

A
AFE
AFG
AFD
AFP
AFV
AFN
ATH
AAL
ACO
ASU
AMA
AMO
FCA
ICO
IYE
AMS
TTP
ppiB
lpxH
ctp
leuA-2
lrp
hyp
cdt
lysR
2.5kb
ppiB: peptidyl-prolyl cis-trans isomerase B
lpxH: UDP-2,3-diacylglucosamine hydrolase
ctp: cation transporting P-type ATPase, putative
leuA-2: 2-isopropyl malate synthase
cdt: c4-dicarboxylate transporter / malic acid transport protein
lysR: transcriptional regulator, Lysr family
B
AFV ACH
AFV SS3
AFN
AMS
tauE
lrp
2.5kb
tauE: Sulfite exporter, TauE/SafE family protein

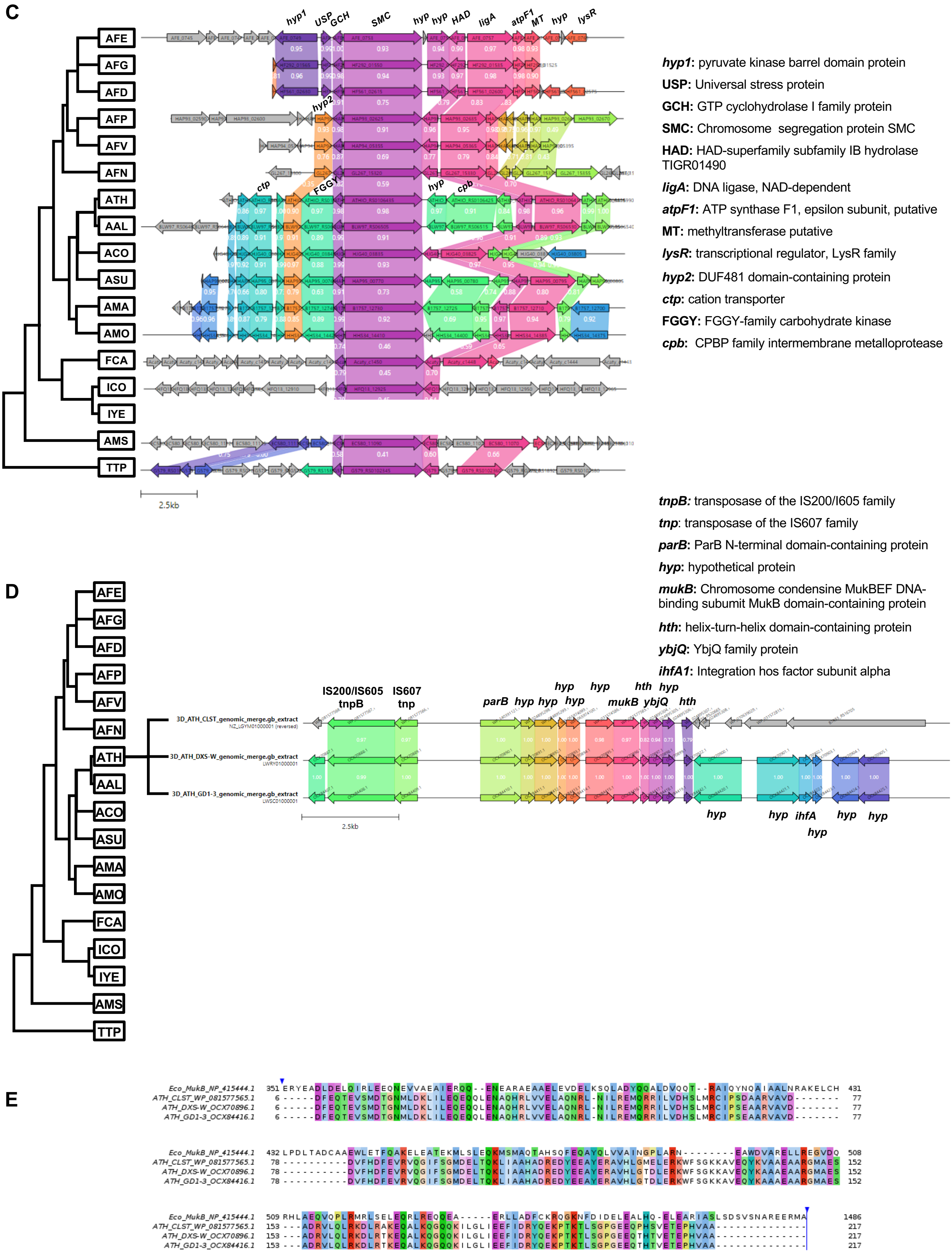
C
AFE
AFG
AFD
AFP
AFV
AFN
ATH
AAL
ACO
ASU
AMA
AMO
FCA
ICO
IYE
AMS
TTP
hyp1
USP
GCH
SMC
hyp
HAD
ligA
atpF1
MT
lysR
hyp2
ctp
FGGY
cpb
2.5kb
hyp1: pyruvate kinase barrel domain protein
USP: Universal stress protein
GCH: GTP cyclohydrolase I family protein
SMC: Chromosome segregation protein SMC
HAD: HAD-superfamily subfamily IB hydrolase TIGR01490
ligA: DNA ligase, NAD-dependent
atpF1: ATP synthase F1, epsilon subunit, putative
MT: methyltransferase putative
lysR: transcriptional regulator, LysR family
hyp2: DUF481 domain-containing protein
ctp: cation transporter
FGGY: FGGY-family carbohydrate kinase
cpb: CPBP family intermembrane metalloprotease
tnpB: transposase of the IS200/I605 family
tnp: transposase of the IS607 family
parB: ParB N-terminal domain-containing protein
hyp: hypothetical protein
mukB: Chromosome condensine MukBEF DNA-binding subumit MukB domain-containing protein
hth: helix-turn-helix domain-containing protein
ybjQ: YbjQ family protein
ihfA1: Integration hos factor subunit alpha
D
IS200/IS605
tnpB
IS607
tnp
parB
mukB
ybjQ
hth
ihfA
3D_ATH_CLST_genomic_merge.gb_extract
NZ_LGYM01000001 (reversed)
3D_ATH_DXS-W_genomic_merge.gb_extract
LWRY01000001
3D_ATH_GD1-3_genomic_merge.gb_extract
LWSC01000001
2.5kb
E
Eco_MukB_NP_415444.1
ATH_CLST_WP_081577565.1
ATH_DXS-W_OCX70896.1
ATH_GD1-3_OCX84416.1

F

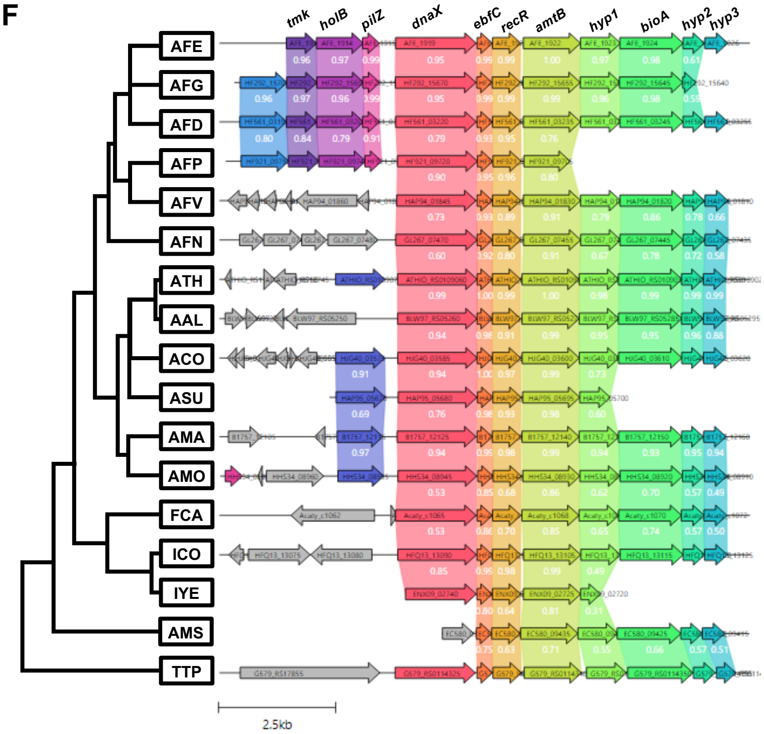

tmk: thymidylate kinase

holB: DNA polymerase III, delta prime subunit

pilZ: type IV pilus assembly protein

dnaX: DNA polymerase III, subunits gamma and tau

amtB: ammonium transporter family protein

hyp1: YjeF-related protein

bioA: adenosylmethionine-8-amino-7-oxononanoate aminotransferase

hyp2: CBS domain protein

hyp3: conserved hypothetical protein TIGR00150

G

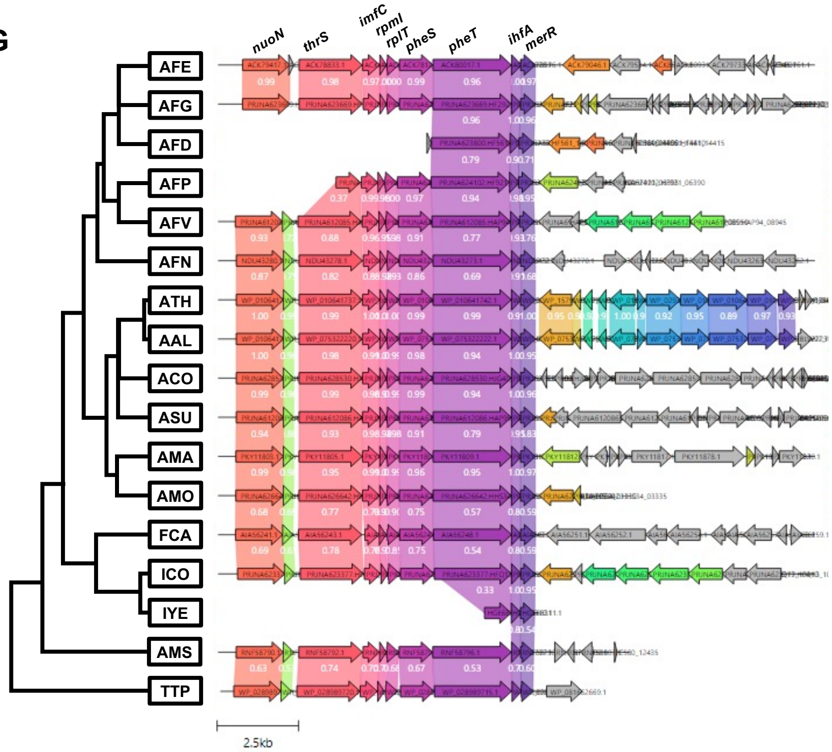

nuoN: NADH-quinone oxidoreductase , subunit N

thrS: threonyl-tRNA synthetase

imfC: translation initiation factor IF-3

rpmL: ribosomal protein L35

rplT: ribosomal protein L20

pheS: phenylalanyl-tRNA synthetase, alpha subunit

pheT: phenylalanyl-tRNA synthetase, beta subunit

merR: transcriptional regulator, MerR family

H

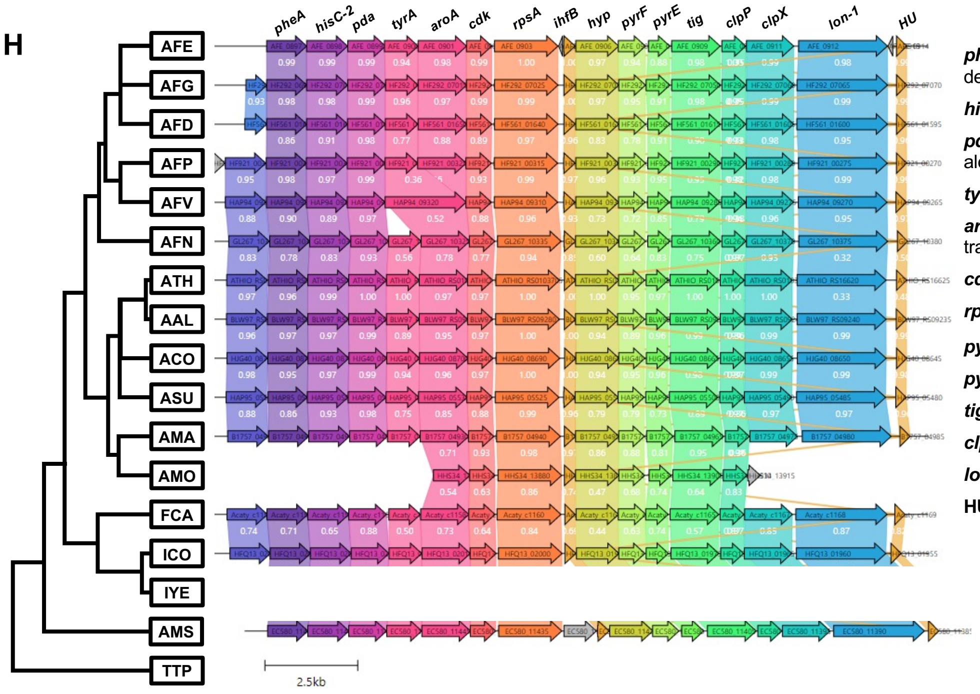

pheA: chorismate mutase/prephenate dehydratase

hisC-2: histidinol-phosphate aminotransferase

pda: phospho-2-dehydro-3-deoxyheptnonate aldolase

tyrA: prephenate dehydrogenase

aroA: 3-phosphoshikimate 1-carboxyvinyl transferase

cdk: cytidylate kinase

rpsA: ribosomal protein S1

pyrF: orotidine 5'-phosphate decarboxylase

pyrE: orotate phosphoribosyl transferase

tig: trigger factor

clpPX: ATP-dependent Clp protease

lon-1: ATP-dependent La protease

HU1: histone-like protein HU *("cluster1")*

I

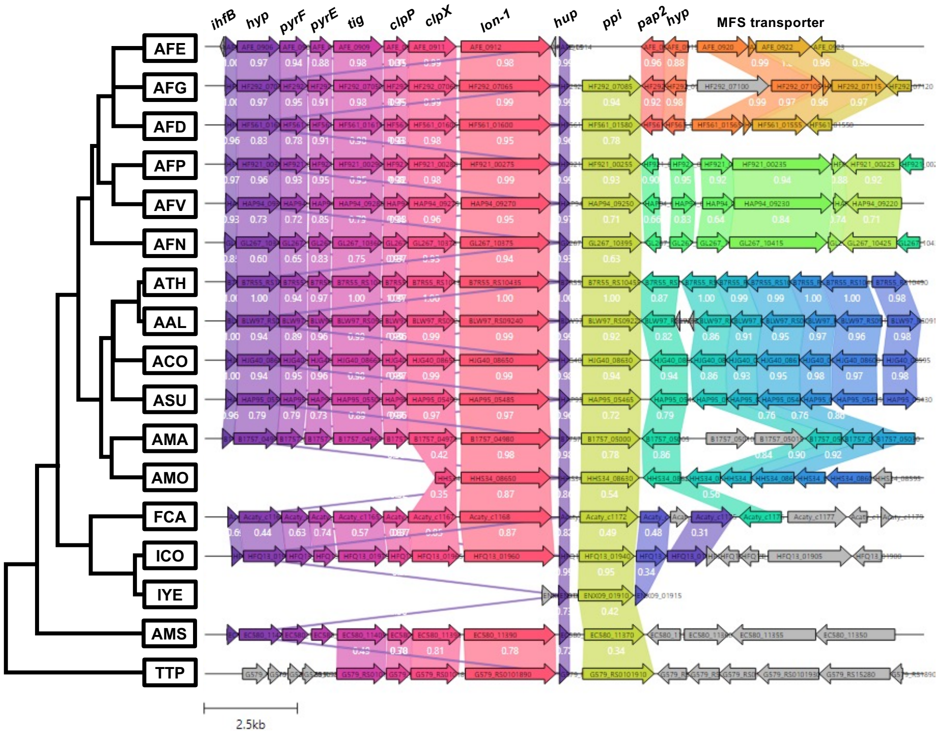

ihfB: integration host factor, beta subunit

pyrF: orotidine 5'-phosphate decarboxylase

pyrE: orotate phosphoribosyl transferase

tig: trigger factor

clpPX: ATP-dependent protease Clp

lon-1: ATP-dependent protease Lat

ppi: peptidyl prolyl isomerase

pap2: PAP2 family protein

J

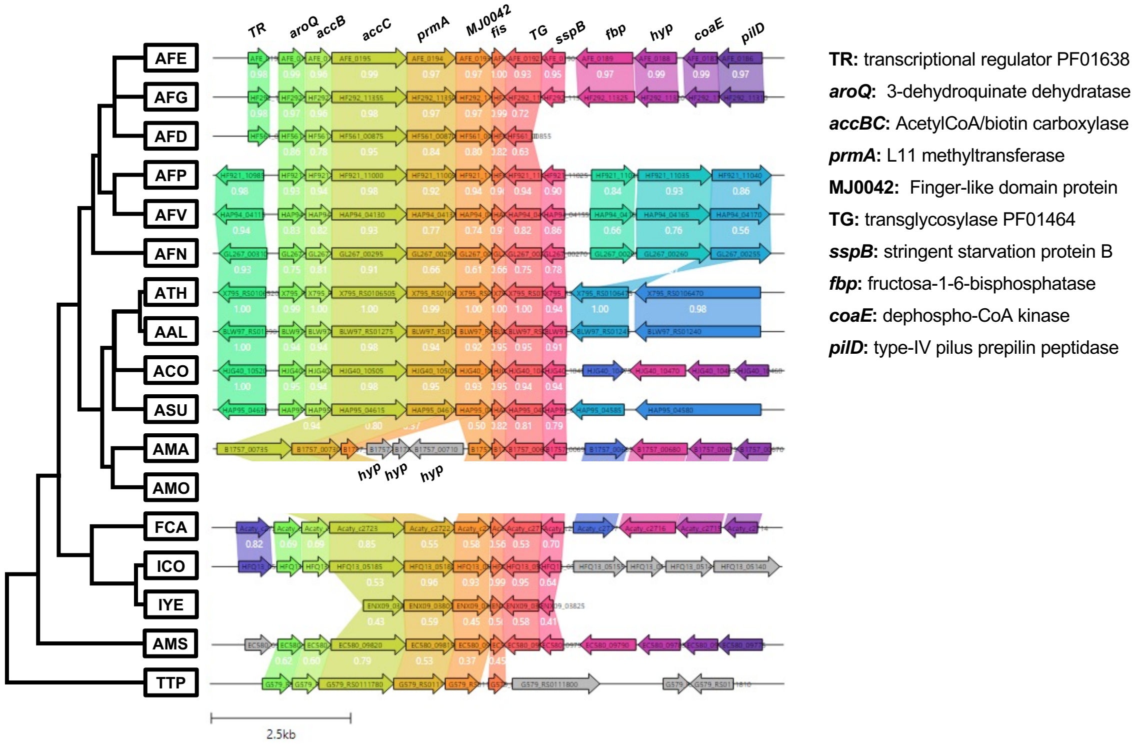

Supplementary Figure 4. Genetic contexts surrounding NAPs protein family in selected *Acidithiobacilllia* class genomes. (**A**) Lrp variant 1. (**B**) Lrp variant 2. (**C**) SMC. (**D**) MukB. (**E**) Aminoacidic sequence alignment of MukB-like NAPs identified in *A. thiooxidans* strains CLST, DXS-W and GD1 (217 aa) and the N-terminal coiled coil region of E. coli MukB (208 aa aligned: aa 356-564, full length E. coli MukB 1486 aa). (**F**) EbfC. (**G**) IHF_A1. (**H**) IHF_B1. (**I**) HU1. (**J**) Fis. Homology based functional assignments of depicted genes is listed in the figure margins. Genetic contexts and similarity scores were calculated using clinker software v0.0.20 (Gilchrist and Chooi, 2021).

Species names and acronyms are as follows: AFE: *Acidithiobacillus ferrooxidans* strains ATCC 23270 or ATCC 53993; AFG, *Acidithiobacillus ferruginosus* strain CF3; AFD, *Acidithiobacillus ferridurans* strains ATCC 33020 or DSM 583; AFP; *Acidithiobacillus ferriphilus* strains DSM 100412 or Malay; AFV, *Acidithiobacillus ferrivorans* strains DSM 2275 or PRJEB5721; AFN, *Acidithiobacillus ferrianus* strain MG; ATH, *Acidithiobacillus thiooxidans* strains ATCC 19377 or A01; AAL, *Acidithiobacillus thiooxidans* subsp. *albertensis* strain DSM 14366; ACO, *Acidithiobacillus concretivorus* strain ATCC 19703; ASU, *Acidithiobacillus sulfurivorans* strain RW2; AMA, *Acidithiobacillus marinus* strain SH; AMO, *Acidithiobacillus monserratensis*, strain GG1-14; FCA, *Fervidacidithiobacilus caldus*, strains ATCC 51756 or DX; ICO, *Igneacidithiobacillus copahuensis* strains VAN18-1 or CV18-3; IYE, *Igneacidithiobacillus yellowstonensis* strain SpSt-908; AMS, *Ambacidithiobacillus sulfuriphilus* strain CJ-2; TTP, *Thermithiobacillus tepidarius* strain DSM 3134.

Extended Figure Legend.

The *smc* gene is found in the immediacy of a GTP cyclohydrolase I family protein coding gene, and in proximity to the genes encoding a HAD-superfamily subfamily IB hydrolase and the DNA ligase NAD-dependent (*ligA*), with lineage specific variations beyond those genes (**Supplementary Figure 4C**). The GTP cyclohydrolase I family protein plays a role in the folate biosynthesis, a key vitamin in *de novo* biosynthesis of DNA precursors. Given that bacterial SMC are involved in the organization and compaction of DNA during chromosome segregation processes (Soppa, 2001), both genes are functionally related to replication and cell division. Other functionally related candidate NAPs were found as part of the *non-conserved* flexible NAP complement in three *A. thiooxidans* strains (CLST, DXS-W and GD1-3; **Supplementary Figure 4D**). These NAPs share sequence similarity to the N-terminal coiled-coil region of the *E. coli* MukB protein (amino acids 356-564), a NAP PF known to contribute to chromosome maintenance or segregation (Weitao et al., 2000; Soppa, 2001). *A. thiooxidans* MukB-like proteins lack the C-terminal domain of *E. coli* MukB protein, essential for MukF and MukE binding during MukFEB complex formation (Yamazoe et al., 1999). The smaller size of *Acidithiobacillia* MukB-like proteins (217 aa versus 1486 aa in *E. coli* MukB, **Supplementary Figure 4E**), together with their restricted occurrence in the class, and the hallmarks of HGT found in their gene vicinity (e.g. genes encoding tranposases, or the plasmid partition protein ParB), argue against a conventional function in maintenance and/or segregation of the chromosome for these NAPs. Interestingly, the three genomes in which the MukB-like proteins are found are among the largest genomes of the class, suggesting that they may have been positively selected to support SMC functions in response to increasing genome sizes, or alternatively to secure MGE maintenance and/or segregation. Both hypotheses remain untested. *Acidithiobacillia* EbfC coding genes vicinities include *recR*, a gene encoding a recombination protein RecR associated with RecBC-independent mechanisms of DNA recombination and repair (Chow and Courcelle, 2004) and *dnaX*, a gene encoding the DNA polymerase III subunit *tau* (**Supplementary Figure 4F**), as has been also previously shown in *Borrelia burdogferi* (Jutras et al., 2012). This suggests EbfC in the acidithiobacilli also plays a role in DNA stabilization during DNA recombinational repair. The *Acidithiobacillia ihfA core* genes are found downstream of the *nuo* operon, encoding the 14 subunits of the NADH:quinone oxidoreductase complex, and in the vicinity of translation-related genes, such as translation initiation factor IF-3 (*imfC*), ribosomal proteins L35 and L20 and threonyl and phenylalanyl-tRNA synthetase coding-genes (**Supplementary Figure 4G**). In turn, the IHF_B *core* protein coding genes are located upstream the core HU1 protein coding genes, in the vicinity of the ribosomal protein S1 gene *rpsA*, and genes related to aromatic amino acids and pyrimidine biosynthesis. They are also located next to genes *clpPX, lon-1* and *ppi,* encoding the ATP-dependent Clp and Lon proteases, respectively (**Supplementary Figure 4H**), and a peptidyl-prolyl isomerase (**Supplementary Figure 4I**). Fis coding genes are located in the proximity of a MJ0042 finger-like domain protein and a ribosomal protein L11 methyltransferase (*prmA*) (**Supplementary Figure 4J**), resembling the conserved genetic organization described for the *E. coli fis* operon (Walker et al., 1999).

References:

Gilchrist, C. L. M., and Chooi, Y.-H. (2020). clinker &clustermap.js: Automatic generation of gene cluster comparison figures. bioRxiv, 2020.11.08.370650. doi: 10.1101/2020.11.08.370650.

Soppa, J. (2001). Prokaryotic structural maintenance of chromosomes (SMC) proteins: distribution, phylogeny, and comparison with MukBs and additional prokaryotic and eukaryotic coiled-coil proteins. *Gene* 278, 253–264. doi: 10.1016/S0378-1119(01)00733-8.

Weitao, T., Dasgupta, S., and Nordström, K. (2000). Role of the mukB gene in chromosome and plasmid partition in Escherichia coli. *Molecular Microbiology* 38, 392–400. doi: https://doi.org/10.1046/j.1365-2958.2000.02138.x.

Yamazoe, M., Onogi, T., Sunako, Y., Niki, H., Yamanaka, K., Ichimura, T., et al. (1999). Complex formation of MukB, MukE and MukF proteins involved in chromosome partitioning in Escherichia coli. *The EMBO Journal* 18, 5873–5884. doi: 10.1093/emboj/18.21.5873.

Chow, K.-H., and Courcelle, J. (2004). RecO Acts with RecF and RecR to Protect and Maintain Replication Forks Blocked by UV-induced DNA Damage in Escherichia coli*. *Journal of Biological Chemistry* 279, 3492–3496. doi: 10.1074/jbc.M311012200.

Jutras, B. L., Bowman, A., Brissette, C. A., Adams, C. A., Verma, A., Chenail, A. M., et al. (2012). EbfC (YbaB) Is a New Type of Bacterial Nucleoid-Associated Protein and a Global Regulator of Gene Expression in the Lyme Disease Spirochete. *Journal of Bacteriology* 194, 3395–3406. doi: 10.1128/JB.00252-12.

Walker, K. A., Atkins, C. L., and Osuna, R. (1999). Functional Determinants of the Escherichia coli fis Promoter: Roles of −35, −10, and Transcription Initiation Regions in the Response to Stringent Control and Growth Phase-Dependent Regulation. *Journal of Bacteriology* 181, 1269–1280. doi: 10.1128/JB.181.4.1269-1280.1999.
