# Supplementary material for: Pangenome-level analysis of nucleoid-associated proteins in the Acidithiobacillia class: insights into their functional roles in mobile genetic elements biology
Source: Front Microbiol. 2023 Sep 25;14:1271138. doi: 10.3389/fmicb.2023.1271138 (PMC10561277; doi:10.3389/fmicb.2023.1271138)
Supplement: Supplementary file 11 [file Data_Sheet_5.PDF]

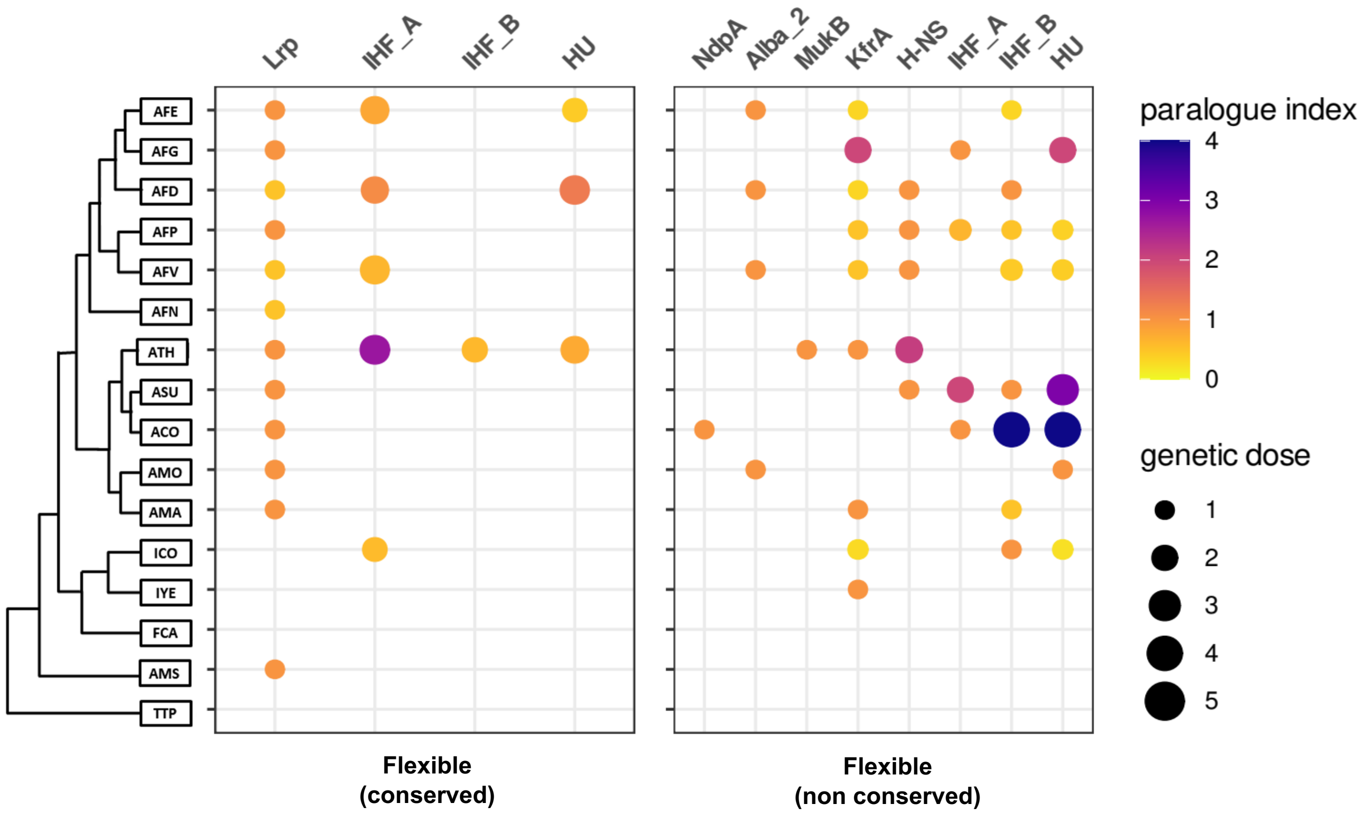

Supplementary Figure 5. Genetic dose and frequency of candidate NAP PFs of the *Acidithiobacillia* class flexible pangenome compartment, classified according to the species. The *paralogue index* was defined as the average genetic dose of each NAP within a given genome, divided by the number of different orthologs of that NAP family in the linage to which that genome belongs. Flexible NAPs correspond to protein families present in < 90% genomes. Distinction between *conserved* (frequency > 0.87 with more than one sequenced genome) and *non-conserved* (frequency ≤ 0.87 or frequency = 1.0 but only one sequenced genome) flexible NAP PFs was made to identify specie-specific conserved NAPs. In order to highlight LRP conservation in *Acidithiobacillus* genus lineages, Lrp proteins were classified as *conserved* flexible protein families.
