# Supplementary material for: Pangenome-level analysis of nucleoid-associated proteins in the Acidithiobacillia class: insights into their functional roles in mobile genetic elements biology
Source: Front Microbiol. 2023 Sep 25;14:1271138. doi: 10.3389/fmicb.2023.1271138 (PMC10561277; doi:10.3389/fmicb.2023.1271138)
Supplement: Supplementary file 12 [file Data_Sheet_6.PDF]

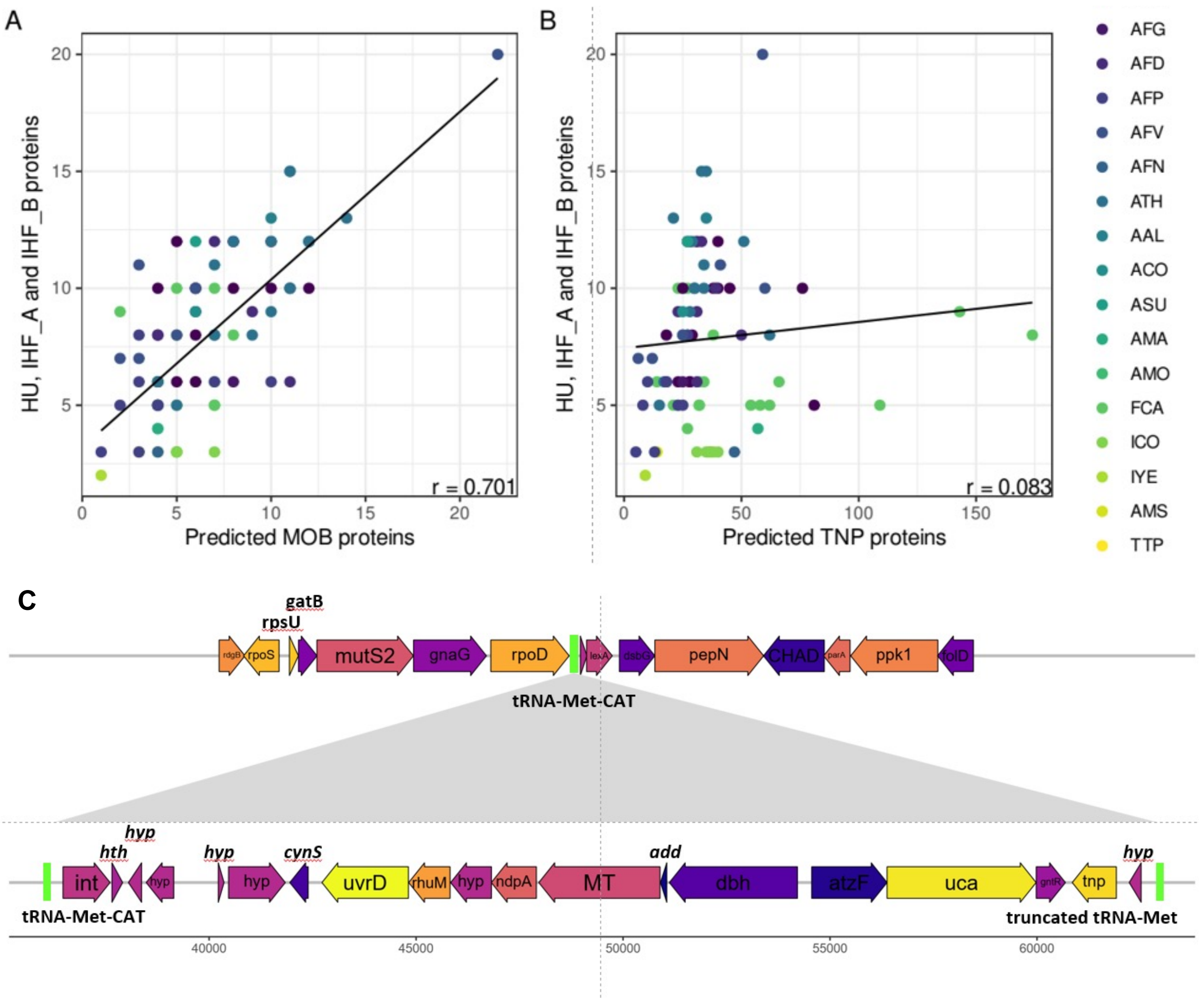

Supplementary Figure 6. Diversified *Acidithiobacillia* flexible NAPs affiliation to the episomal and/or integrative mobilome. **(A)** Correlation between total NAPs numbers or between HU and IHF NAPs numbers and CONJScan predicted MOB proteins of the following types MOBP, MOBF, MOBQ, MOBH or MOBV (Guglielmini et al., 2014), or (**B**) TNPpred predicted transposases found in *Acidithiobacillia* class genomes. Pearson's correlation coefficient (*r*) is shown at bottom right of each scatter plot. (**C**) Genetic context of exclusive *ndpA* in *A. sulfurivorans* RW2 within a predicted MGE integrated in the chromosome at tRNA-Met-CAT. Genes depicted in the scheme are the following: *rpsU*, 30S ribosomal protein S21; *gatB*, GatB/YqeY domain-containing protein; *mutS2*, endonuclease MutS2; *gnaG*, DNA primase; *rpoD*, RNA polymerase sigma factor RpoD; *int*, site-specific integrase; *hth*, helix-turn-helix domain-containing protein; *hyp*, hypothetical protein; *cynS*, cyanase; *uvrD*, UvrD-helicase domain-containing protein; *rhuM*, virulence RhuM family protein; *ndpA*, nucleoid-associated protein; MT, site-specific DNA-methyltransferase; *add*, addiction module protein; *dbh*, DEAD/DEAH box helicase family protein (pseudogene); *atzF*, allophanate hydrolase; *uca*, urea carboxylase; *gntR*, GntR family transcriptional regulator; *tnp*, transposase; *hyp*, hypothetical protein; *hyp*, hypothetical protein; *lexA*, SOS-response repressor and protease LexA; *hyp*, hypothetical protein; *aapN*, membrane alanine aminopeptidase N; *adcY*, Adenylate cyclase; *parA*, ParA-like protein, *ppk*, polyphosphate kinase.

Species names and acronyms are as follows: AFE: *Acidithiobacillus ferrooxidans*; AFG, `*Acidithiobacillus ferruginosus*´; AFD, *Acidithiobacillus ferridurans*; AFP, *Acidithiobacillus ferriphilus*; AFV, *Acidithiobacillus ferrivorans*; AFN, *Acidithiobacillus ferrianus*; ATH, *Acidithiobacillus thiooxidans*; AAL, *Acidithiobacillus thiooxidans* subsp. *albertensis*; ACO, `*Acidithiobacillus concretivorus*´; ASU, `*Acidithiobacillus sulfurivorans*´; AMA, `*Acidithiobacillus marinus*´; AMO, `*Acidithiobacillus monserratensis*´; FCA, `*Fervidacidithiobacilus caldus*´; ICO, `*Igneacidithiobacillus copahuensis*´; IYE, `*Igneacidithiobacillus yellowstonensis*´; AMS, `*Ambacidithiobacillus sulfuriphilus*´; TTP, *Thermithiobacillus tepidarius*.

References:

Guglielmini, J., Néron, B., Abby, S. S., Garcillán-Barcia, M. P., la Cruz, F. de, and Rocha, E. P. C. (2014). Key components of the eight classes of type IV secretion systems involved in bacterial conjugation or protein secretion. Nucleic Acids Research 42, 5715–5727. doi: 10.1093/nar/gku194.
