# Supplementary material for: Pangenome-level analysis of nucleoid-associated proteins in the Acidithiobacillia class: insights into their functional roles in mobile genetic elements biology
Source: Front Microbiol. 2023 Sep 25;14:1271138. doi: 10.3389/fmicb.2023.1271138 (PMC10561277; doi:10.3389/fmicb.2023.1271138)
Supplement: Supplementary file 13 [file Data_Sheet_7.PDF]

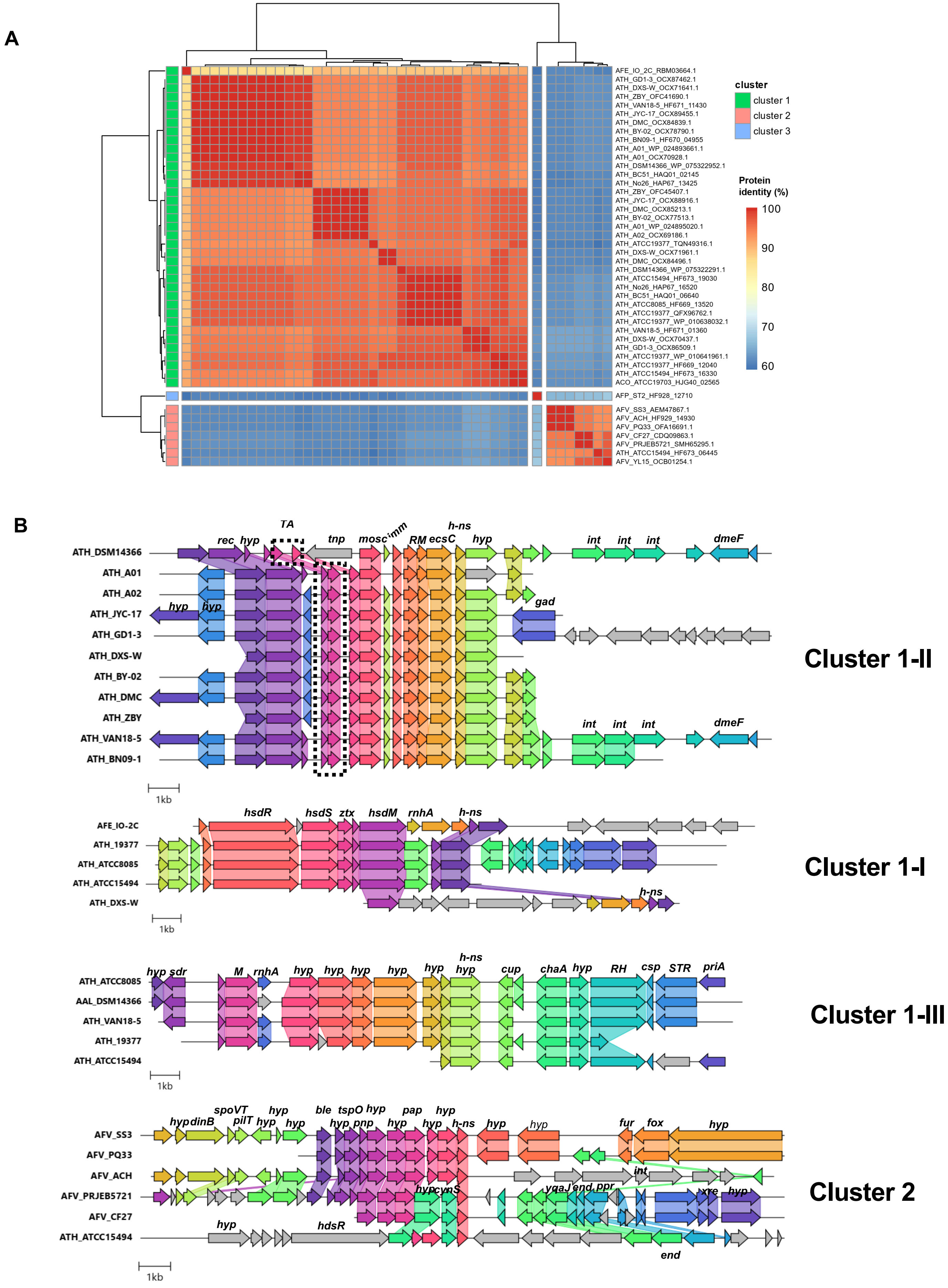

A
cluster
cluster 1
cluster 2
cluster 3
Protein identity (%)
100
90
80
70
60
AFE_IO_2C_RBM03664.1
ATH_GD1-3_OCX87462.1
ATH_DXS-W_OCX71641.1
ATH_ZBY_OFC41690.1
ATH_VAN18-5_HF671_11430
ATH_JYC-17_OCX89455.1
ATH_DMC_OCX84839.1
ATH_BY-02_OCX78790.1
ATH_BN09-1_HF670_04955
ATH_A01_WP_024893661.1
ATH_A01_OCX70928.1
ATH_DSM14366_WP_075322952.1
ATH_BC51_HAQ01_02145
ATH_No26_HAP67_13425
ATH_ZBY_OFC45407.1
ATH_JYC-17_OCX88916.1
ATH_DMC_OCX85213.1
ATH_BY-02_OCX77513.1
ATH_A01_WP_024895020.1
ATH_A02_OCX69186.1
ATH_ATCC19377_TQN49316.1
ATH_DXS-W_OCX71961.1
ATH_DMC_OCX84496.1
ATH_DSM14366_WP_075322291.1
ATH_ATCC15494_HF673_19030
ATH_No26_HAP67_16520
ATH_BC51_HAQ01_06640
ATH_ATCC8085_HF669_13520
ATH_ATCC19377_QFX96762.1
ATH_ATCC19377_WP_010638032.1
ATH_VAN18-5_HF671_01360
ATH_DXS-W_OCX70437.1
ATH_GD1-3_OCX86509.1
ATH_ATCC19377_WP_010641961.1
ATH_ATCC19377_HF669_12040
ATH_ATCC15494_HF673_16330
ACO_ATCC19703_HJG40_02565
AFP_ST2_HF928_12710
AFV_SS3_AEM47867.1
AFV_ACH_HF929_14930
AFV_PQ33_OFA16691.1
AFV_CF27_CDQ09863.1
AFV_PRJEB5721_SMH65295.1
ATH_ATCC15494_HF673_06445
AFV_YL15_OCB01254.1
B
ATH_DSM14366
ATH_A01
ATH_A02
ATH_JYC-17
ATH_GD1-3
ATH_DXS-W
ATH_BY-02
ATH_DMC
ATH_ZBY
ATH_VAN18-5
ATH_BN09-1
rec hyp
TA
tnp
mosc mm
h-ns
RM ecsC
hyp
int int int
dmeF
hyp hyp
gad
int int int
dmeF
1kb
Cluster 1-II
AFE_IO-2C
ATH_19377
ATH_ATCC8085
ATH_ATCC15494
ATH_DXS-W
hsdR hsdS ztx hsdM rnhA h-ns
h-ns
1kb
Cluster 1-I
ATH_ATCC8085
AAL_DSM14366
ATH_VAN18-5
ATH_19377
ATH_ATCC15494
hyp sdr
M rnhA
hyp hyp hyp hyp
hyp
h-ns
hyp
cup
chaA hyp
RH
csp
STR
priA
1kb
Cluster 1-III
AFV_SS3
AFV_PQ33
AFV_ACH
AFV_PRJEB5721
AFV_CF27
ATH_ATCC15494
spoVT
hyp dinB
pilT hyp
hyp
hyp
ble tspO hyp
hyp pnp
pap
hyp
hyp
hyp h-ns
hyp
hyp
fur
fox
hyp
int
hyp cynS
yqaJ end ppr
xre hyp
hyp
hdsR
end
1kb
Cluster 2

Supplementary Figure 7. NAPs of the H-NS protein family recovered from *Acidithiobacillia* class genomes. **(A)** Clustering analysis of H-NS proteins based on pairwise blast identity percentages, shown as heatmap. Proteins were clustered based on their pairwise BlastP identity values, and colored according to the color bar in the figure margin. Clusters 1 and 2 correspond to H-NS1 and H-NS2 protein variants. Subvariants identified in cluster 1 are labels with Roman numbers. (**B**) Genetic contexts surrounding conserved H-NS1 and H-NS2 protein variants in selected *Acidithiobacilllia* class genomes. Contexts are depicted for selected representatives of clusters 1-I, 1-II, 1-III and 2, as labeled.
