# Supplementary material for: Pangenome-level analysis of nucleoid-associated proteins in the Acidithiobacillia class: insights into their functional roles in mobile genetic elements biology
Source: Front Microbiol. 2023 Sep 25;14:1271138. doi: 10.3389/fmicb.2023.1271138 (PMC10561277; doi:10.3389/fmicb.2023.1271138)
Supplement: Supplementary file 14 [file Data_Sheet_8.PDF]

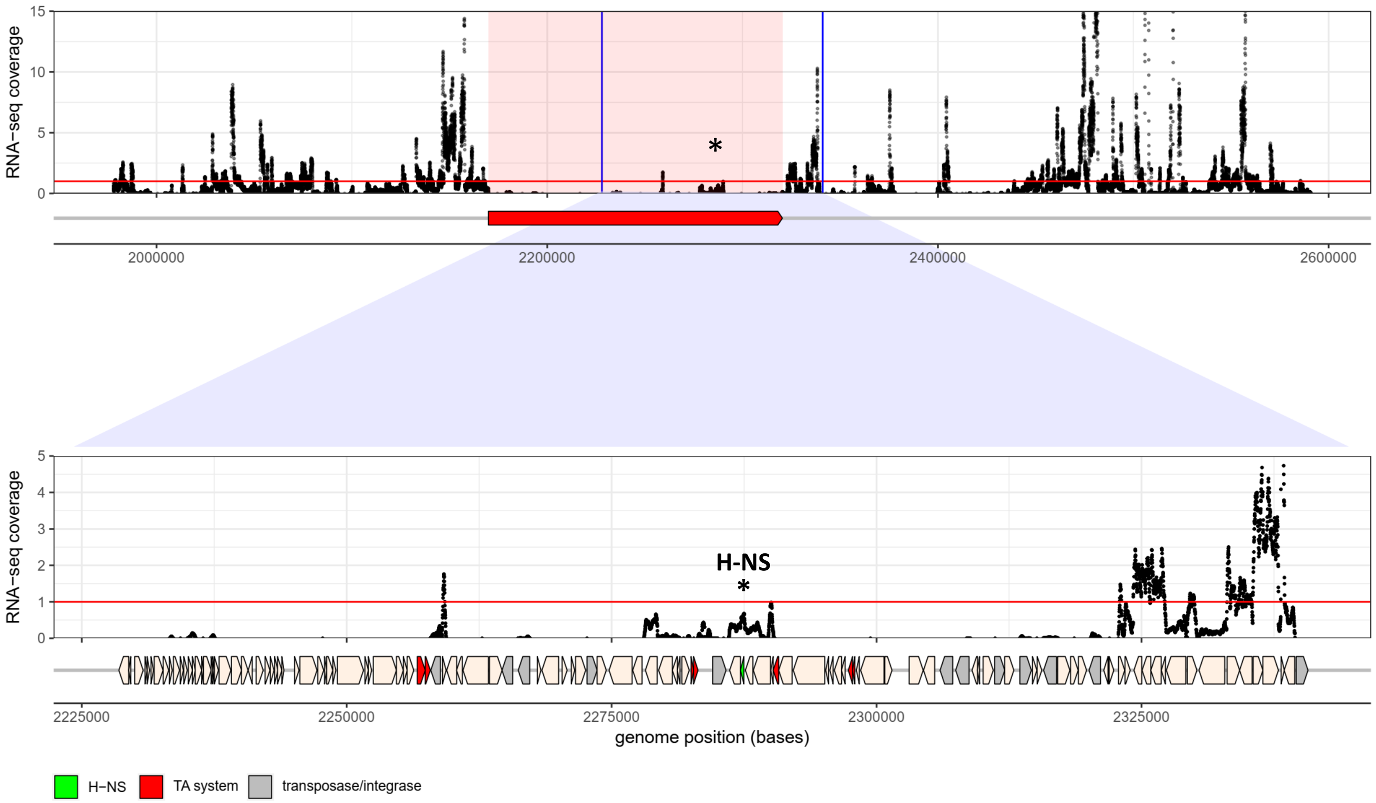

Supplementary Figure 8. Relative expression levels in genomic regions around H-NS coding gene in *Acidithiobacillus thiooxidans* type strain ATCC 19377. RNA-seq expression profiles for *A. thiooxidans* ATCC 19377 grown in elemental sulfur at pH 2.5 (BioProject PRJNA541131) were downloaded from SRA database (https://www.ncbi.nlm.nih.gov/sra). Selected runs (SRR9016879, SRR9016878 and SRR9016873) were aligned to *A. thiooxidans* ATCC 19377 reference genome (NZ_CP045571) and RNA-seq coverage was calculated as the number of reads per bin (10 base-long) and normalized by reads per genomic content (1x normalization). *Upper panel.* Asterisks shows the location of H-NS coding gene and a predicted MGE is shown as a red box. *Bottom panel.* Enlargement of the region encompassing H-NS and part of the predicted MGE delimited in blue, showing relative expression levels and predicted coding genes. H-NS (green), toxin-antitoxin (TA) systems (red) and transposases/integrases (grey) coding genes are highlighted. Note that an RNA-seq coverage of 1.0 represent the whole genome coverage average.
